# Body ownership and the four-hand illusion

**DOI:** 10.1038/s41598-018-19662-x

**Published:** 2018-02-01

**Authors:** Wen-Yeo Chen, Hsu-Chia Huang, Yen-Tung Lee, Caleb Liang

**Affiliations:** 1 Graduate Institute of Brain and Mind Sciences, National Taiwan University, Taipei, Taiwan; 2 Department of Philosophy, National Taiwan University, Taipei, Taiwan

## Abstract

Recent studies of the rubber hand illusion (RHI) have shown that the sense of body ownership is constrained by several factors and yet is still very flexible. However, exactly how flexible is our sense of body ownership? In this study, we address this issue by investigating the following question: is it possible that one may have the illusory experience of owning four hands? Under visual manipulation, the participant adopted the experimenter’s first-person perspective (1PP) as if it was his/her own. Sitting face to face, the participant saw four hands—the experimenter’s two hands from the adopted 1PP together with the subject’s own two hands from the adopted third-person perspective (3PP). We found that: (1) the four-hand illusion did not occur in the passive four-hand condition. (2) In the active four-hand condition, the participants tapped their index fingers, imitated by the experimenter. When tactile stimulations were not provided, the key illusion was not induced, either. (3) Strikingly, once all four hands began to act with the same pattern and received synchronous tactile stimulations at the same time, many participants felt as if they had two more hands. These results show that the sense of body ownership is much more flexible than most researchers have suggested.

## Introduction

The sense of body ownership is related to whether a body part or a whole body is experienced as *mine*. Investigations on the rubber hand illusion^1^ (RHI) and full-body illusions^2–4^ (FBI) have greatly enhanced our understanding of this basic form of bodily self-consciousness. Inspired by the standard RHI and FBI paradigms, two rather different but complementary lines of research have emerged that motivated this current study. The first tries to ascertain how flexible our sense of body ownership can be. Previous literature has shown that, by manipulating visual and tactile stimulations, subjects could feel as if they owned two right hands^5,6^, or two left hands^7^. In another set-up, a healthy subject can experience the illusion of having an invisible hand^8^. In the case of FBI, flexibility is observed as well: participants felt as if they were positioned outside their body^3^, or someone else’s body was their own^9^, or they had two bodies in virtual reality circumstances^10^. All of these studies suggest that our sense of body ownership is highly flexible.

In contrast, the second line of research aims to articulate the constraints that would hinder a relevant illusion. For example, researchers have shown that the RHI would be abolished if the multisensory stimulations were asynchronous, or if the size of the seen fake hand was smaller than the participant’s real hand^11,12^. The RHI is also considered as constrained by anatomical, postural and spatial factors^13,14^. For example, it has been suggested that the illusion would not happen if the viewed object does not look like a hand^15^, if the fake hand was rotated 90° or 180° with respect to the subject^6,13^, or if the fake hand was placed too far away from the subject^16^. A mismatch in the brushing directions on the subject’s hand and the rubber hand would also hinder the illusion^17^.

On the one hand, the above two lines of research are complementary in the sense that both flexibility and constraint are important aspects of the sense of body ownership; studying one of them helps to illuminate the other. On the other hand, there are some conflicting results that require further investigations. For example, Tsakiris regards postural congruency as a pre-requisite for the RHI, and maintains that “If there is incongruence between the posture of felt and seen hands, the seen hand will not be experienced as part of one’s own body”^14^ (p. 708). However, in our previous study on the “self-touching illusion”^18^, we challenged this constraint by manipulating the participant’s visual perspective and introducing a participant-experimenter interaction. Sitting face to face, the subject and experimenter held a paintbrush with their right hand to brush each other’s left hand, such that the subject was touching someone and being touched at the same time, as well as watching via a head-mounted display (HMD) his/her own body in front of him/herself. Using this set-up, we induced an illusory sense of body ownership where the virtual body was viewed from an adopted 3PP (“It felt as if I was brushing my own hand”, “It felt as if the body in front of me was mine”). We showed that postural incongruence can sometimes be overcome by visual form congruence and what we call “body agency” (the subject feels his/her own active movement via proprioception) and “visual agency” (the active movement that the participant *sees* via an HMD)^18^ (pp. 8–9).

**Figure 1:**
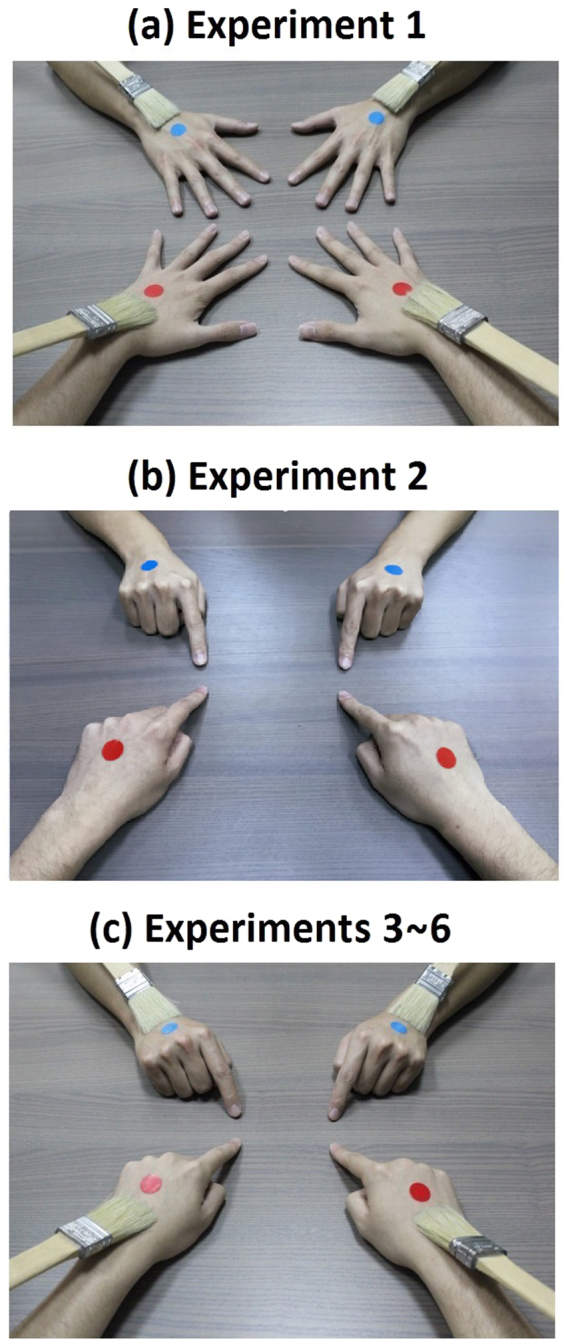
Images in Experiments 1~6 seen via HMD. (**a**) Experiment 1. The participant saw two pairs of hands via the HMD: the experimenter’s hands from the adopted 1PP with red tags, and the participant’s own hands from the adopted 3PP (180° reverse) with blue tags. The participant passively received tactile stimulations either synchronously or asynchronously. (**b**) Experiment 2. The participant saw the experimenter’s hands from the adopted 1PP with red tags, and the participant’s own hands from the adopted 3PP (180° reverse) with blue tags. Both the participant and the experimenter tapped their index fingers without receiving tactile stimulations. (**c**) Experiments 3~6. In Experiments 3 and 4, the experimenter’s hands were seen from the adopted 1PP with red tags, and the participant’s own hands were seen from the adopted 3PP (180° reverse) with blue tags. In Experiments 5 and 6, the experimenter’s hands were viewed from the adopted 3PP (180° reverse) with blue tags, and the participant’s own hands were viewed from the adopted 1PP with red tags. Both the participant and the experimenter tapped their index fingers and received tactile stimulations. To measure SCR, two single-use foam electrodes were attached to the inner side of the participant’s left palm. The wires were carefully put under the participant’s arm. So both the electrodes and the wires would not be seen by the participant via the HMD.

In this study, we investigated the following issue: Is it possible to overcome postural incongruence such that healthy subjects may experience ownership of hands from both 1PP and 3PP at the same time? Based on the findings about the “self-touching illusion”, we think that it is likely that the sense of body ownership is even more malleable than it is suggested by the current literature. We designed a series of experiments that tried to push the RHI paradigm one step forward: the passive four-hand condition (Experiment 1, Fig. 1a), active four-hand condition (without touch) (Experiment 2, Fig. 1b) and the active four-hand condition (with touch) (Experiments 3~6, Fig. 1c). Using an HMD, we created a “four-hand” situation by combining the adopted 1PP and the adopted 3PP. Then we added in both body agency and visual agency as key factors. Based on the RHI literature, we suspected that a variant of RHI could be induced on the hands viewed from the adopted 1PP. Regarding the hands seen from the adopted 3PP, we surmised that postural incongruence could be jointly overcome by body agency, visual agency and a high degree of visual form congruence. Therefore, we hypothesized that it is possible to induce a “four-hand illusion” in the active—but not in the passive—condition such that the participants felt as if they possessed an additional pair of hands. If so, this would show that the sense of body ownership is much more flexible than most researchers think.

## Results

For Experiments 1~3, the median values with interquartile ranges (IQRs) of the questionnaire statements and the skin conductance response (SCR) values are shown in Table 1. A Passive two-hand condition was conducted in support of Experiment 1 (see Supplementary Information). The data of Experiments 4~6 are presented in Table 2.

**Table 1:** Median Values and interquartile ranges (IQRs) of questionnaire statements and SCR in Experiments 1, 2, and 3.

questionnaires	Experiment 1 Median (IQR)		Experiment 2 Median (IQR)		Experiment 3 Median (IQR)	
	Sync.	Async.	Sync.	Async.	Sync.	Async.
1. It felt as if the hands with red tags were mine.	1 (−2~1)	−2 (−3~−0.5)	−1 (−2~1)	−3 (−3~−1)	2 (0~3)	−2 (−2~−0.5)
2. It felt as if the hands with blue tags were mine.	1 (0~2)	2 (1~3)	2 (0~2)	2 (1.5~2)	2 (0~2)	2 (1.5~3)
3. The touches that I felt were located on the hands with red tags.	1 (0~2)	−2 (−2~0)			2 (1~2)	−2 (−2~1)
4. The touches that I felt were located on the hands with blue tags.	−1 (−1~1)	1 (−1~2)			1 (−1.5~2)	2 (0~2.5)
5. It felt as if I could control the hands with red tags.	−1 (−2~1.5)	−2 (−3~−1)	−1 (−3~0.5)	−2 (−3~−1)	1 (−0.5~3)	−2 (−2~−1)
6. It felt as if I could control the hands with blue tags.	2 (−1~2)	2 (1~3)	2 (0.5~3)	2 (1~3)	2 (0~2)	2 (2~3)
7. At a certain point, it felt as if I had two more hands.	−1 (−2~1)	−2 (−2.5~1)	0 (−2~1)	−2 (−3~1)	2 (−0.5~2)	−2 (−2~0)
8. I felt that my hands were brushed.	3 (3~3)	3 (2~3)	−3 (−3~−2)	−3 (−3~−2)	3 (3~3)	3 (2.5~3)
SCR on 1PP-hands	2.9 (1.9~4.5)	1.5 (0.7~3.2)	1.4 (0.8~3.6)	1.2 (0.6~2.6)	4.6 (2.8~5.9)	2.0 (1.2~4.0)
SCR on 3PP-hands	2.2 (1.3~2.6)	1.8 (1.1~3.9)	1.3 (0.4~2.6)	1.2 (0.4~2.2)	2.3 (1.5~3.4)	3.3 (2.7~5.9)

### Experiment 1: Passive four-hand condition

**Figure 2:**
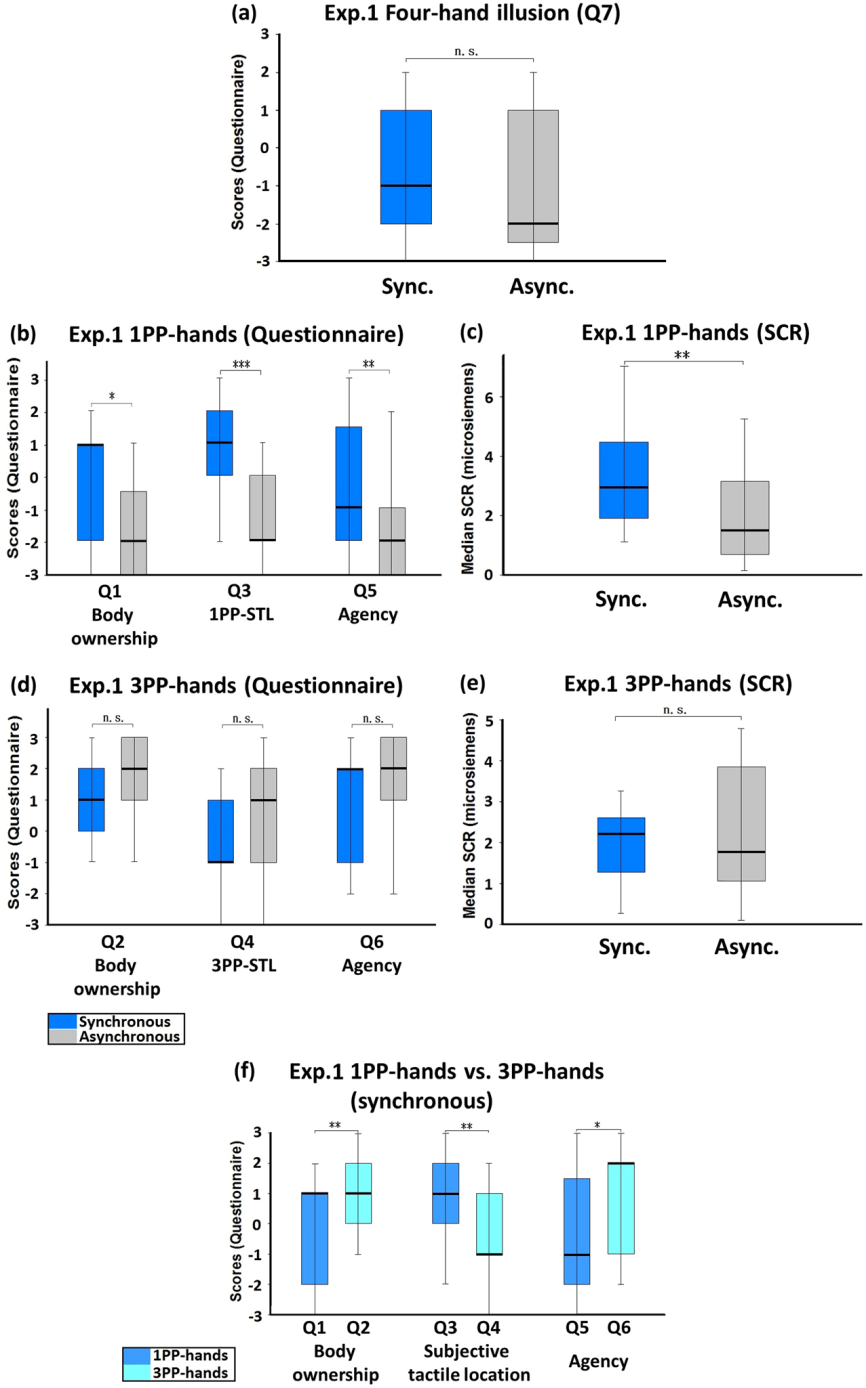
Experiment 1: Passive four-hand condition. (**a**) Questionnaire results regarding the four-hand illusion (Q7). Both medians of Q7 in the synchronous and the asynchronous conditions were negative and the comparison showed no significant difference, indicating that there was no four-hand illusion. (**b**) Questionnaire results regarding 1PP-hands. The ratings regarding body ownership (Q1), 1PP-subjective tactile location (Q3), and agency (Q5) were significantly higher in the synchronous than in the asynchronous condition, but both of the medians of Q5 were negative. (**c**) SCR results regarding 1PP-hands. SCR was measured when the 1PP-hands were threatened with a knife. A significant difference was observed between the synchronous and the asynchronous conditions. (**d**) Questionnaire results regarding 3PP-hands. The ratings regarding body ownership (Q2), 3PP-subjective tactile location (Q4) and agency (Q6) were lower in the synchronous than in the asynchronous condition, but showed no significant difference. (**e**) SCR results regarding 3PP-hands. SCR was measured when the 3PP-hands were threatened with a knife. No significant difference existed between the synchronous and the asynchronous conditions. (**f**) Comparison between 1PP-hands and 3PP-hands in the synchronous condition. There were significant differences between the ratings of 1PP-hands and 3PP-hands regarding body ownership, subjective tactile location, and agency. Bold lines indicate the medians; upper and lower limits of the box plot indicate the 75^th^ and 25^th^ percentile. The error bars represent the whole range of the ratings of the statement. Significance levels: *p < 0.05; **p < 0.01; ***p < 0.001. Abbreviation: STL, subjective tactile location.

In Experiment 1, the participant watched via the HMD the experimenter’s hands (with red tags) from the adopted 1PP and the participant’s own hands (with blue tags) from the adopted 3PP, and received tactile stimulations (Fig. 1a). The “red-tag statements” (Q1, Q3, Q5) measured the participants’ subjective feelings regarding the 1PP-hands. The “blue-tag statements” (Q2, Q4, Q6) addressed the *3PP-hands* (the hands viewed from the adopted 3PP). The four-hand illusion was not observed in this experiment (Q7, Z = −1.525, p = 0.127; Fig. 2a). The scores of the red-tag statements were significantly higher in the synchronous condition for body ownership (Q1), subjective tactile location (Q3), and agency (Q5), than in the asynchronous condition (Q1: Z = −2.521, p = 0.012; Q3: Z = −3.856, p < 0.001; Q5: Z = −2.621, p = 0.009; Fig. 2b). Regarding the SCR that was measured when the 1PP-hands (the experimenter’s hands) were threatened, the synchronous condition was significantly higher than the asynchronous condition (Z = −2.960, p = 0.003; Fig. 2c). However, the induced body ownership regarding the 1PP-hands (Q1) in the synchronous condition was significantly weaker than that in Passive two-hand condition (Z = −2.476, p = 0.013; see Supplementary Information). Although the median scores of the blue-tag statements in the synchronous condition were lower than those in the asynchronous condition, there was no significant difference between them (Q2: Z = −1.081, p = 0.280; Q4: Z = −1.772, p = 0.076; Q6: Z = −1.362, p = 0.173; Fig. 2d). Regarding the SCR that was measured when the 3PP-hands (the participant’s hands) were threatened, the synchronous condition showed no significant difference from the asynchronous condition (Z = −0.955, p = 0.339; Fig. 2e). The control statement Q8 showed no significant difference, either (Z = −1.633, p = 0.102).

We observed a significant difference between Q2 (ownership of 3PP-hands) and Q1 (ownership of 1PP-hands) in the synchronous condition (Z = −2.775, p = 0.006, Fig. 2f). Of the 25 subjects, 18 (72%) answered positively (+1, +2 or +3) that the 3PP-hands were theirs. When comparing Q2 in the synchronous condition of Experiment 1 with Q1 in the synchronous condition of Passive two-hand condition, there was no significant difference between them (Z = −0.110, p = 0.913). These results indicate that, in the synchronous passive four-hand condition, the participant felt that the 3PP-hands were his/her own hands. The median of Q3 was significantly higher than that of Q4 in the synchronous condition (Z = −2.694, p = 0.007, Fig. 2f), indicating that the participant felt as if the tactile sensations he/she felt were located in the 1PP-hands rather than in the 3PP-hands. The score of Q6 was significantly higher than that of Q5 in the synchronous condition (Z = −2.366, p = 0.018, Fig. 2f), suggesting that the subjects felt that they could control the 3PP-hands.

### Experiment 2: Active four-hand condition (without touch)

**Figure 3:**
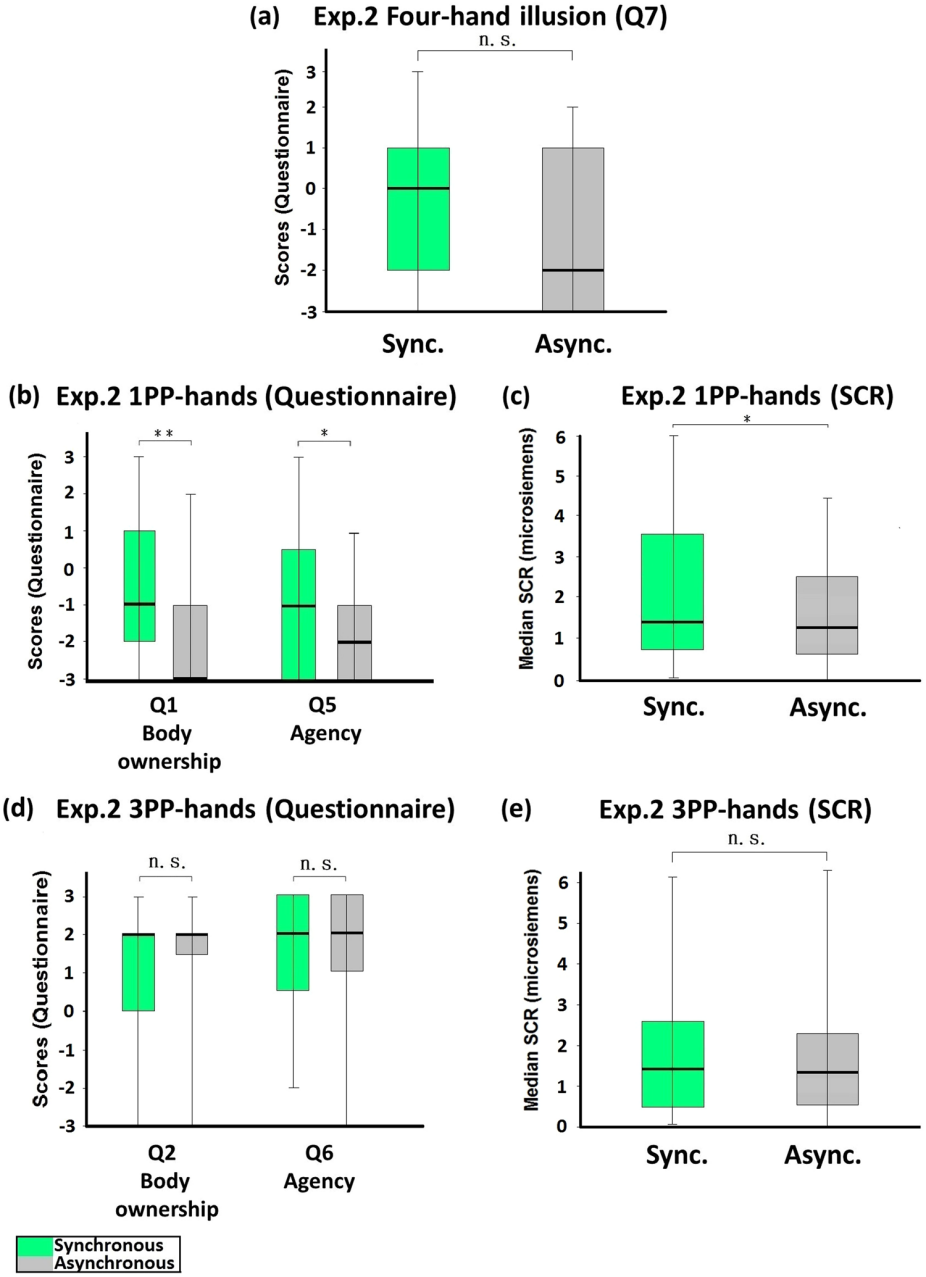
Experiment 2: Active four-hand condition (without touch). (**a**) Questionnaire results regarding the four-hand illusion (Q7). The medians of Q7 in both the synchronous and the asynchronous conditions were less than positive one (+1) and the comparison showed no significant difference, indicating that there was no four-hand illusion. (**b**) Questionnaire results regarding 1PP-hands. The ratings regarding body ownership (Q1) and agency (Q5) were significantly higher in the synchronous than in the asynchronous condition, but the medians were negative. (**c**) SCR results regarding 1PP-hands. SCR was measured when the 1PP-hands were threatened with a knife. A significant difference was observed between the synchronous and the asynchronous conditions. (**d**) Questionnaire results regarding 3PP-hands. The ratings regarding body ownership (Q2) and agency (Q6) were higher than +1, and there was no significant difference between the synchronous and the asynchronous conditions. (**e**) SCR results regarding 3PP-hands. SCR was measured when the 3PP-hands were threatened with a knife. No significant difference existed between the synchronous and the asynchronous conditions. Bold lines indicate the medians; upper and lower limits of the box plot indicate the 75^th^ and 25^th^ percentile. The error bars represent the whole range of the ratings of the statement. Significance levels: *p < 0.05; **p < 0.01; ***p < 0.001. Abbreviation: STL, subjective tactile location.

In Experiment 2, the participant tapped both of his/her index fingers, while seeing four hands tapping with index fingers either synchronously or asynchronously (Fig. 1b). Since tactile stimulations were not provided, Q3 and Q4 did not apply in this experiment. The medians of Q7 (four-hand illusion) were less than +1 in both the synchronous and the asynchronous conditions (Fig. 3a), so there was no four-hand illusion. The medians of the red-tag statements in the synchronous condition were also low, even though they were significantly higher than the asynchronous condition (Q1: Z = −2.977, p = 0.003; Q5: Z = −2.159, p = 0.031; Fig. 3b). The SCR measured when the 1PP-hands were threatened was significantly higher in the synchronous condition than in the asynchronous condition (Z = −2.301, p = 0.021, Fig. 3c). The medians of the blue-tag statements showed no significant difference between the synchronous and the asynchronous conditions (Q2: Z = −1.183, p = 0.237; Q6: Z = −1.141, p = 0.254; Fig. 3d), so as the SCR results of the 3PP-hands (Z = −1.197, p = 0.231, Fig. 3e).

### Experiment 3: Active four-hand condition (with touch)

**Figure 4:**
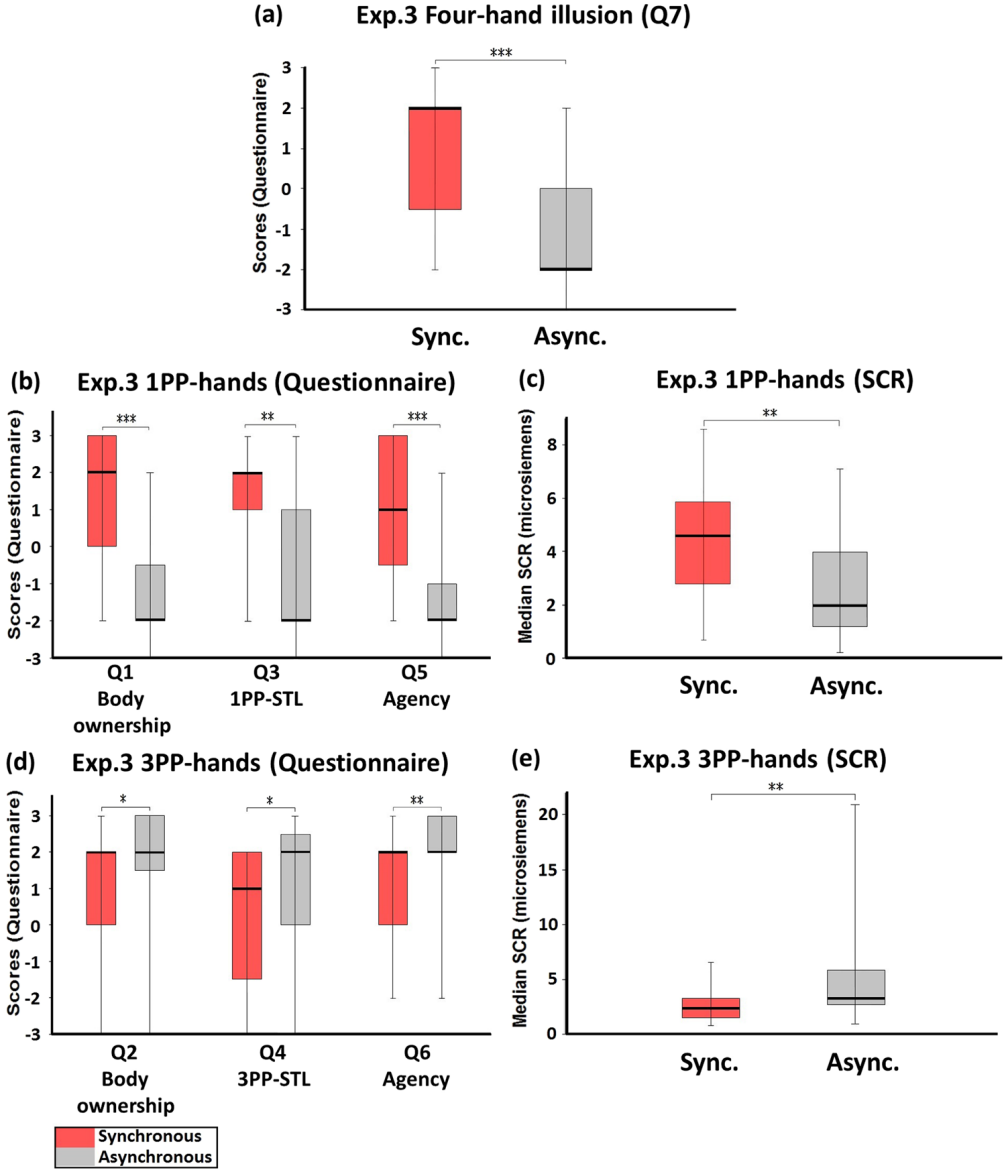
Experiment 3: Active four-hand condition (with touch). (**a**) Questionnaire results regarding the four-hand illusion (Q7). The ratings regarding the four-hand illusion were significantly higher in the synchronous than in the asynchronous condition. (**b)** Questionnaire results regarding 1PP-hands. The ratings regarding body ownership (Q1), 1PP-subjective tactile location (Q3) and agency (Q5) were significantly higher in the synchronous than in the asynchronous condition. (**c**) SCR results regarding 1PP-hands. SCR was measured when the 1PP-hands were threatened with a knife. There was a significant difference between the synchronous and the asynchronous conditions. (**d**) Questionnaire results regarding 3PP-hands. The ratings regarding body ownership (Q2), 3PP-subjective tactile location (Q4), and agency (Q6) were significantly lower in the synchronous condition than in the asynchronous condition. (**e**) SCR results regarding 3PP-hands. SCR was measured when the 3PP-hands were threatened with a knife. There was a significant difference between the synchronous and the asynchronous conditions. Bold lines indicate the medians; upper and lower limits of the box plot indicate the 75th and 25th percentile. The error bars represent the whole range of the ratings of the statement. Significance levels: *p < 0.05; **p < 0.01; ***p < 0.001. Abbreviation: STL, subjective tactile location.

In Experiment 3, the participant tapped both of his/her index fingers and received tactile stimulations, while seeing four hands tapping with index fingers (Fig. 1c). The medians of Q7 (four-hand illusion) were significantly higher in the synchronous condition than in the asynchronous condition (Z = −3.612, p < 0.001; Fig. 4a). It is here that we observed the four-hand illusion. During the synchronous condition, 17 participants (68%) answered positively (+1, +2 or +3) that they had *two more hands*.

The medians of the red-tag statements were significantly higher in the synchronous condition than in the asynchronous condition (Q1: Z = −3.767, p < 0.001; Q3: Z = −2.995, p = 0.003; Q5: Z = −3.622, p < 0.001; Fig. 4b). The SCR measured when the 1PP-hands were threatened was also significantly higher in the synchronous condition than in the asynchronous condition (Z = −3.323, p = 0.001, Fig. 4c). Q1 in the synchronous condition of Experiment 3 was significantly higher than that in Experiment 1 (Z = −2.478, p = 0.013; see Supplementary Information). These data indicate that the subject felt as if the 1PP-hands were his/her hands. The scores for the blue-tag statements were significantly lower in the synchronous condition than in the asynchronous condition (Q2: Z = −2.132, p = 0.033; Q4: Z = −2.061, p = 0.039; Q6: Z = −2.776, p = 0.005; Fig. 4d). Control statement Q8 showed no significant difference between the synchronous and the asynchronous conditions (Z = −1.633, p = 0.102). The corresponding SCR that reflected the threat to the 3PP-hands was significantly lower in the synchronous condition than in the asynchronous condition (Z = −2.677, p = 0.007, Fig. 4e). We think this was due to the fact that the subjects saw their own hands from the 3PP.

**Figure 5:**
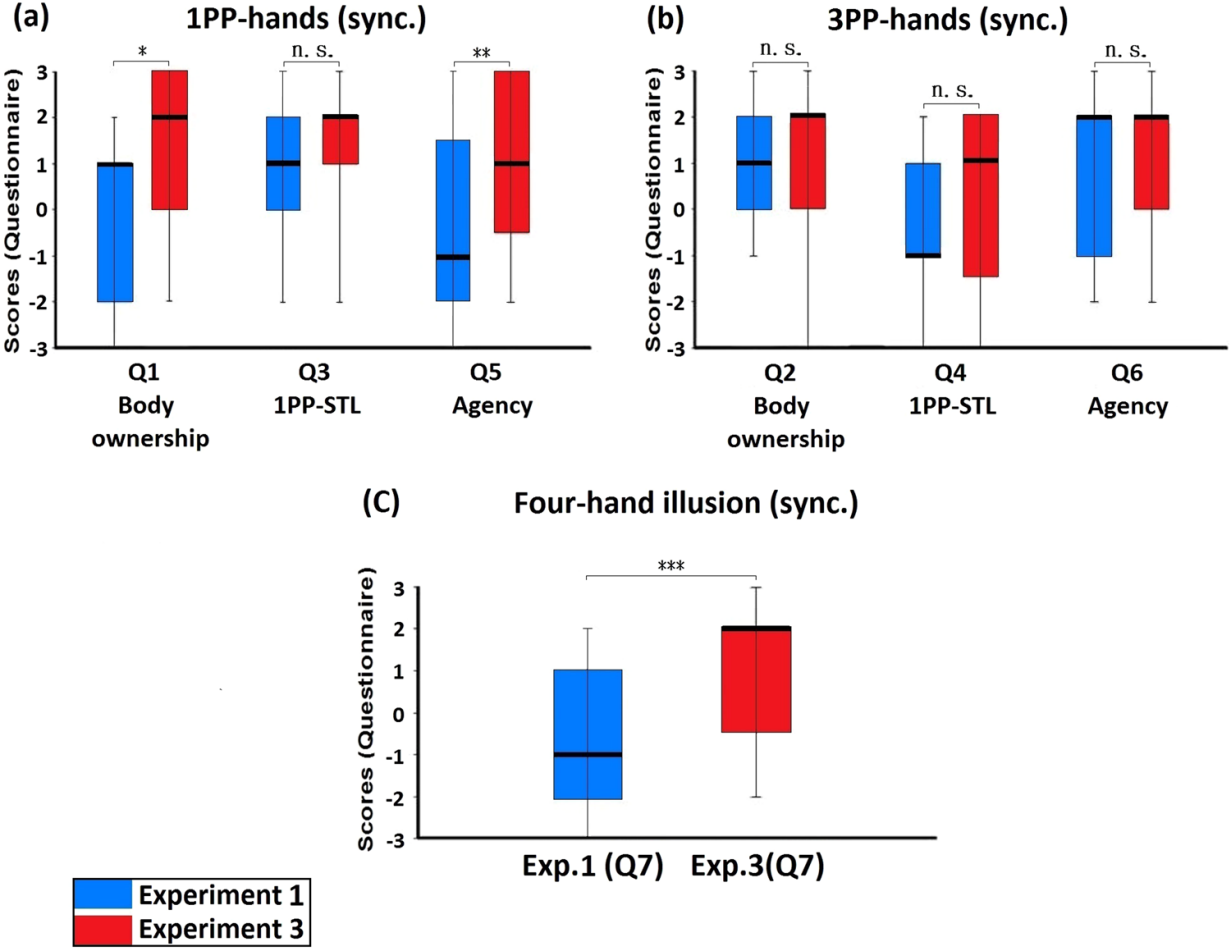
Comparisons between Experiment 1 and Experiment 3. (**a**) Comparison regarding 1PP-hands. The ratings of 1PP-hands in Experiment 3 regarding body ownership (Q1) and agency (Q5) were significantly higher than that in Experiment 1; the ratings regarding 1PP-subjective tactile location (Q3) showed no significant differences. (**b**) Comparison regarding 3PP-hands. The ratings of 3PP-hands in Experiment 3 regarding body ownership (Q2), 3PP-subjective tactile location (Q4) and Agency (Q6) showed no significant differences compared with Experiment 1. (**c**) Comparison regarding the four-hand illusion. The ratings of the four-hand illusion (Q7) in Experiment 3 were significantly higher than that in Experiment 1. Bold lines indicate the medians; upper and lower limits of the box plot indicate the 75^th^ and 25^th^ percentile. The error bars represent the whole range of the ratings of the statement. Significance levels: *p < 0.05; **p < 0.01; ***p < 0.001. Abbreviation: STL, subjective tactile location.

On the one hand, we think the data of the blue-tag statements suggest that the subject’s experience of the 3PP-hands was influenced by his/her experience of the 1PP-hands in the synchronous but not in the asynchronous condition, and that seeing both the 1PP-hands and the 3PP-hands in one view was a subjectively integrated experience. On the other hand, in addition to the fact that the 3PP-hands were the subject’s own hands, statistical comparisons suggest that the score of Q2 in the synchronous condition still indicates the ownership of the 3PP-hands. First, the sense of 3PP-hand ownership (Q2) was slightly (but not significantly) stronger in the synchronous condition of Experiment 3 than that of Experiment 1 (Fig. 5b). As a supplement to this, the sense of 3PP-hand ownership in the synchronous condition of Experiment 3 was significantly stronger than the sense of 1PP-hand ownership (Q1) in the synchronous condition of Experiment 1 (Z = −2.453, p = 0.014). Second, we found that the sense of agency in the 3PP-hands (Q6) in the synchronous condition of Experiment 3 was as strong as that in the synchronous condition of Experiment 1 (Fig. 5b). Q6 in the synchronous condition of Experiment 3 was also significantly higher than Q5 in the synchronous condition of Experiment 1 (Z = −2.750, p = 0.006). Since Q5 in the synchronous condition of Experiment 1 indicated no sense of agency, this difference supports that the participants felt the sense of agency with regard to the 3PP-hands. Together, these data suggest that in the synchronous condition of Experiment 3 the participants felt as if the 3PP-hands were their own hands and as if they could control them.

When comparing the synchronous condition of Experiment 3 with that of Experiment 1, we found the following: regarding the 1PP-hands, the scores of body ownership (Q1) and agency (Q5) were significantly higher in Experiment 3 than in Experiment 1 (Q1: Z = −2.478, p = 0.013; Q5: Z = −2.795, p = 0.005), but the score of subjective tactile location (Q3) showed no such difference (Q3: Z = −0.661, p = 0.509; Fig. 5a). Regarding the 3PP-hands, the scores of body ownership (Q2), subjective tactile location (Q4) and agency (Q6) showed no significant difference between Experiment 3 and Experiment 1 (Q2: Z = −0.121, p = 0.904; Q4: Z = −1.167, p = 0.243; Q6: Z = −0.299, p = 0.765; Fig. 5b). Most importantly, the score of four-hand illusion (Q7) in Experiment 3 was significantly higher than in Experiment 1 (Z = −3.519, p < 0.001; Fig. 5c).

**Figure 6:**
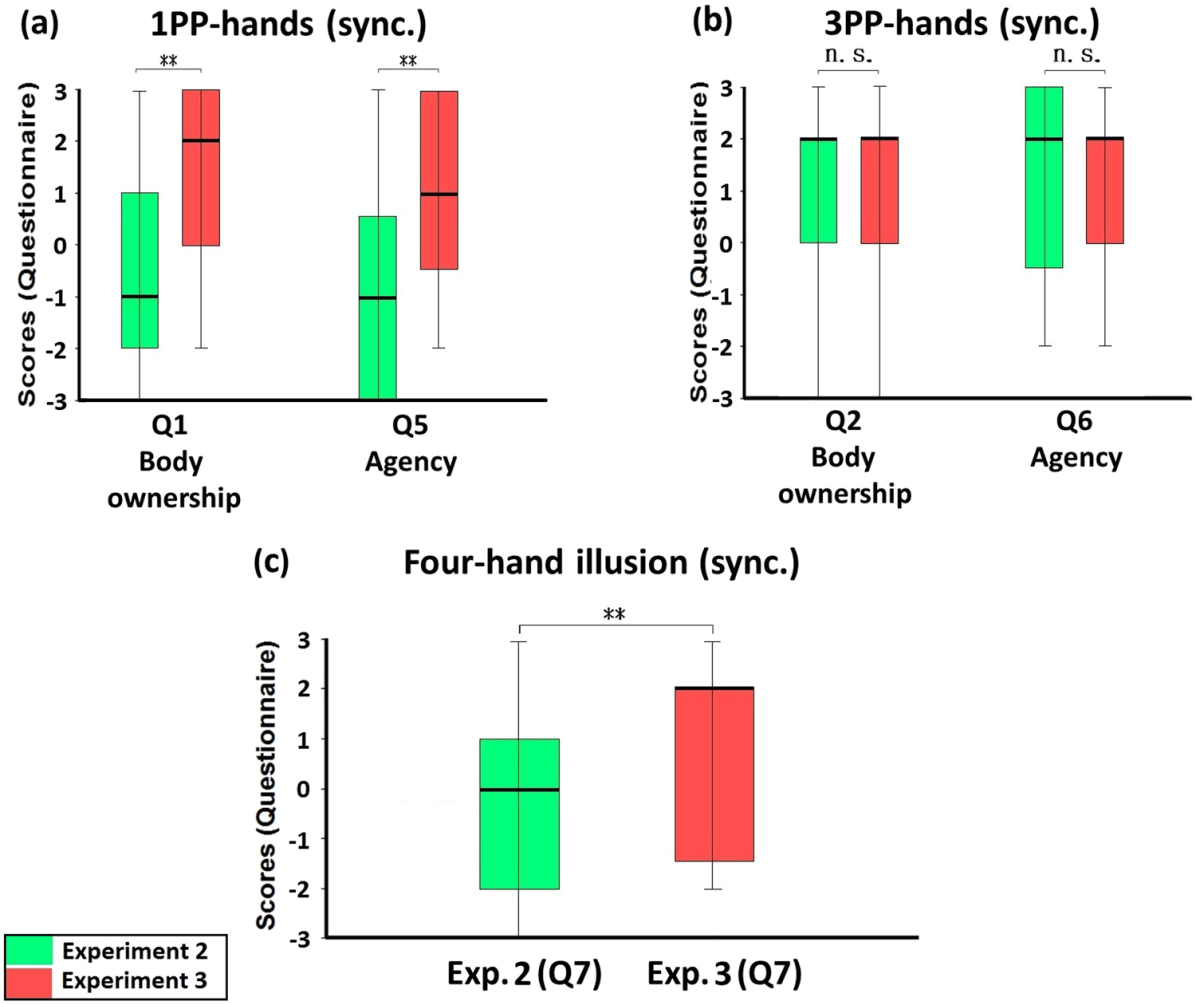
Comparisons between Experiment 2 and Experiment 3. (**a**) Comparison regarding 1PP-hands. The ratings of the 1PP-hands in Experiment 3 regarding body ownership (Q1) and agency (Q5) were significantly higher than that in Experiment 2. (**b**) Comparison regarding 3PP-hands. The ratings of 3PP-hands in Experiment 3 regarding body ownership (Q2) and Agency (Q6) showed no significant differences compared with Experiment 2. (**c**) Comparison regarding the four-hand illusion. The ratings of the four-hand illusion (Q7) in Experiment 3 were significantly higher than that in Experiment 2, indicating that the four-hand illusion occurred only in the active four-hand condition with synchronous tactile stimulations. Bold lines indicate the medians; upper and lower limits of the box plot indicate the 75^th^ and 25^th^ percentile. The error bars represent the whole range of the ratings of the statement. Significance levels: *p < 0.05; **p < 0.01; ***p < 0.001. Abbreviation: STL, subjective tactile location.

Finally, when we compared the synchronous condition of Experiment 3 with that of Experiment 2, we found that the scores of body ownership (Q1) and agency (Q5) of the 1PP-hands were significantly higher in Experiment 3 than in Experiment 2 (Q1: Z = −3.066, p = 0.002; Q5: Z = −3.191, p = 0.001; Fig. 6a). Regarding the 3PP-hands, the scores of body ownership (Q2) and agency (Q6) showed no significant difference between Experiment 3 and Experiment 2 (Q2: Z = −0.527, p = 0.598; Q6: Z = −0.928, p = 0.353; Fig. 6b). In particular, the score of four-hand illusion (Q7) in Experiment 3 was significantly higher than in Experiment 2 (Z = −3.036, p = 0.002; Fig. 6c).

### Control active four-hand conditions: Experiments 4~6

#### Experiment 4

**Figure 7:**
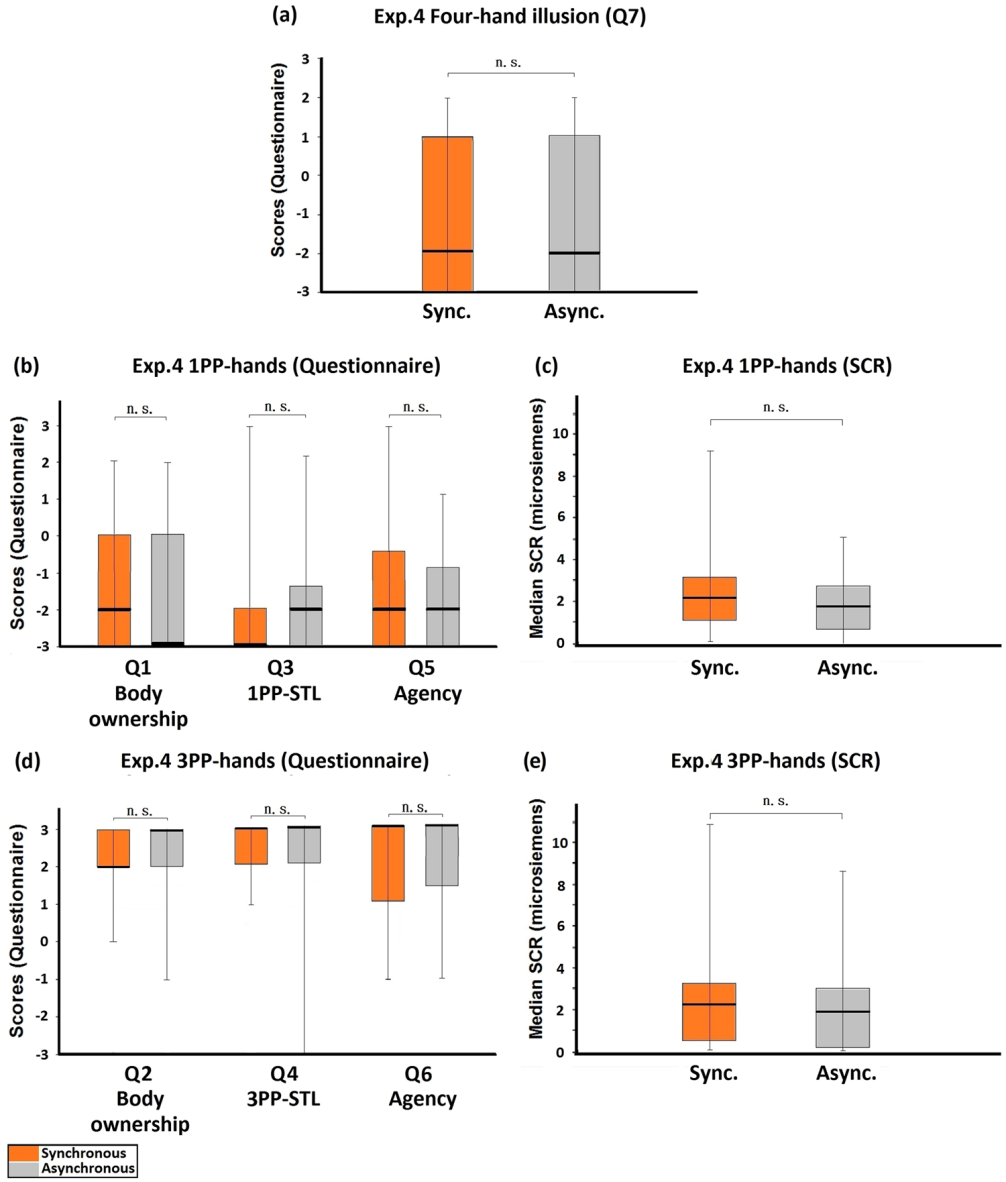
Experiment 4: Control active four-hand condition. (**a**) Questionnaire results regarding the four-hand illusion (Q7). Both medians of Q7 in the synchronous and the asynchronous conditions were negative and the comparison showed no significant difference, indicating that there was no four-hand illusion. (**b**) Questionnaire results regarding 1PP-hands. The ratings regarding body ownership (Q1), 1PP-subjective tactile location (Q3), and agency (Q5) showed no significant differences and all the medians were rather low. (**c**) SCR results regarding 1PP-hands. SCR was measured when the 1PP-hands were threatened with a knife. The SCR values were not different between the synchronous and the asynchronous conditions. (**d**) Questionnaire results regarding 3PP-hands. The medians regarding body ownership (Q2), 3PP-subjective tactile location (Q4) and agency (Q6) were all very high and showed no significant difference. (**e**) SCR results regarding 3PP-hands. SCR was measured when the 3PP-hands were threatened with a knife. No significant difference existed between the synchronous and the asynchronous conditions. Bold lines indicate the medians; upper and lower limits of the box plot indicate the 75^th^ and 25^th^ percentile. The error bars represent the whole range of the ratings of the statement. Significance levels: *p < 0.05; **p < 0.01; ***p < 0.001. Abbreviation: STL, subjective tactile location.

The procedure of Experiment 4 was the same as Experiment 3, except that the brushing was asynchronous in both the synchronous and the asynchronous conditions. The four-hand illusion was not induced in this experiment (Q7, Z = −0.315, p = 0.753; Fig. 7a). The medians of the red-tag statements showed no significant difference between the synchronous and the asynchronous conditions (Q1: Z = −0.845, p = 0.398; Q3: Z = −0.634, p = 0.526; Q5: Z = −0.971, p = 0.332; Fig. 7b), so as the rest of the data: the SCR measured when the 1PP-hands were threatened (Z = −1.776, p = 0.076, Fig. 7c), the scores for the blue-tag statements (Q2: Z = −0.638, p = 0.523; Q4: Z = −0.277, p = 0.782; Q6: Z = −0.160, p = 0.873; Fig. 7d), the SCR that reflected the threat to the 3PP-hands (Z = −1.857, p = 0.063; Fig. 7e), and the control statement Q8 (Z = −0.447, p = 0.655).

**Table 2:** Median Values and interquartile ranges (IQRs) of questionnaire statements and SCR in Experiments 4, 5, and 6.

questionnaires	Experiment 4 Median (IQR)		Experiment 5 Median (IQR)		Experiment 6 Median (IQR)	
	Sync.	Async.	Sync.	Async.	Sync.	Async.
1. It felt as if the hands with red tags were mine.	−2 (−3~0)	−3 (−3~0)	3 (2~3)	3 (3~3)	3 (2~3)	3 (2.5~3)
2. It felt as if the hands with blue tags were mine.	2 (2~3)	3 (2~3)	−2 (−2.5~0.5)	−3 (−3~−2)	−1 (−2.5~−0.5)	−3 (−3~−2)
3. The touches that I felt were located on the hands with red tags.	−3 (−3~−2)	−2 (−3~−1.5)	3 (3~3)	3 (3~3)	3 (2~3)	3 (2~3)
4. The touches that I felt were located on the hands with blue tags.	3 (2~3)	3 (2~3)	−3 (−3~−2)	−3 (−3~−2)	−3 (−3~−1.5)	−3 (−3~−2)
5. It felt as if I could control the hands with red tags.	−2 (−3~−0.5)	−2 (−3~−1)	3 (3~3)	3 (3~3)	3 (2~3)	3 (2~3)
6. It felt as if I could control the hands with blue tags.	3 (1~3)	3 (1.5~3)	−2 (−2~−0.5)	−3 (−3~−2)	−1 (−2~1)	−2 (−3~−2)
7. At a certain point, it felt as if I had two more hands.	−2 (−3~1)	−2 (−3~1)	−1 (−2.5~0.5)	−3 (−3~−1.5)	−1 (−2~0.5)	−2 (−3~−1)
8. I felt that my hands were brushed.	3 (3~3)	3 (3~3)	3 (3~3)	3 (3~3)	3 (2.5~3)	3 (3~3)
SCR on 1PP-hands	2.2 (1~3.0)	1.5 (0.7~2.4)	4.1 (2.1~6.2)	4.6 (2.4~6.2)	3.9 (2.4~5.2)	3.1 (2.1~5.3)
SCR on 3PP-hands	2.2 (0.6~3.2)	1.7 (0.2~2.9)	3.1 (1.5~6.1)	1.7 (0.7~2.5)	2.3 (1.4~3.7)	2.0 (0.9~2.8)

#### Experiment 5

**Figure 8:**
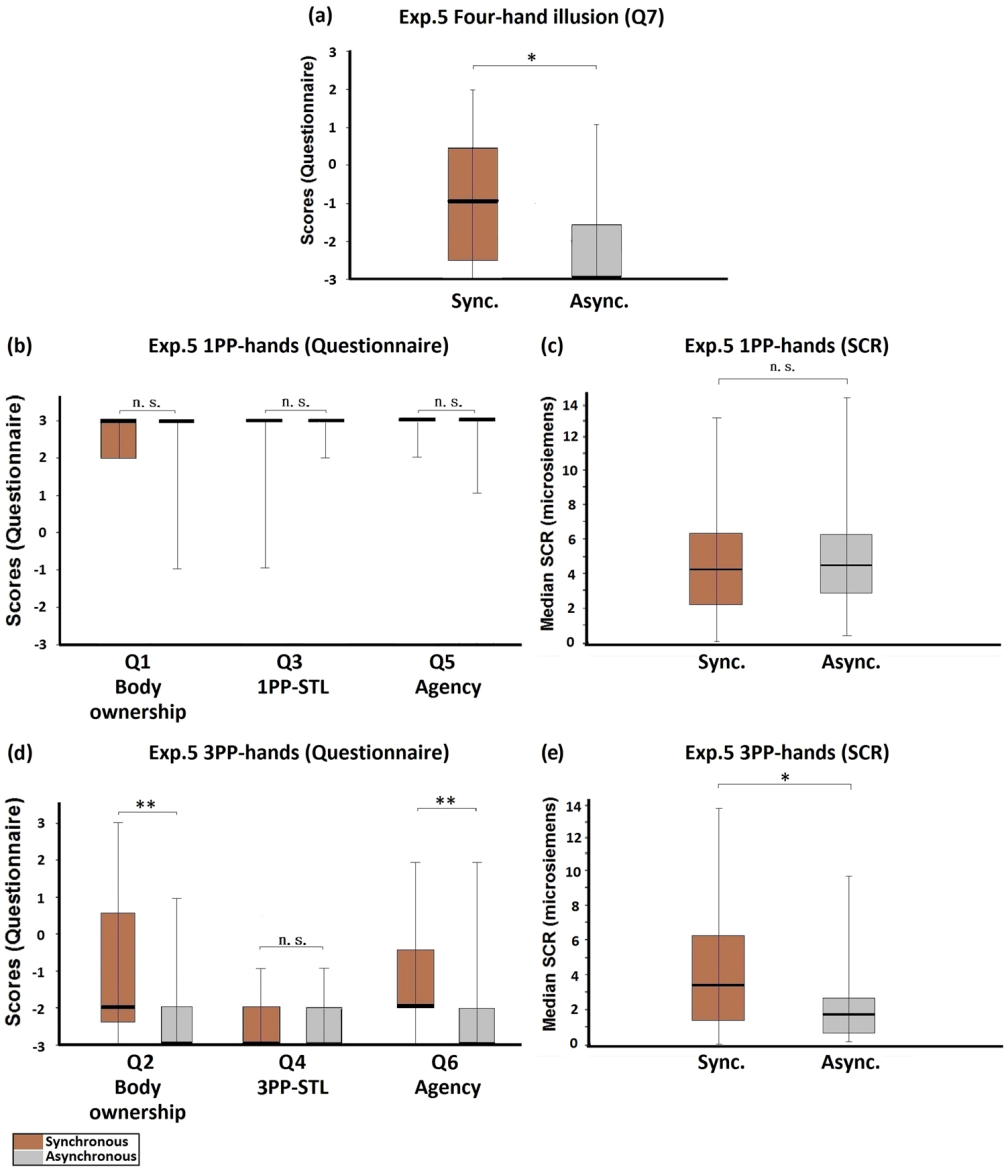
Experiment 5: Control active four-hand condition. (**a**) Questionnaire results regarding the four-hand illusion (Q7). Although a significant difference was observed between the synchronous and the asynchronous conditions, the medians in both conditions were negative. (**b**) Questionnaire results regarding 1PP-hands. The ratings regarding body ownership (Q1), 1PP-subjective tactile location (Q3) and agency (Q5) were quite high and no significant differences existed. (**c**) SCR results regarding 1PP-hands. SCR was measured when the 1PP-hands were threatened with a knife. There was no significant difference between the synchronous and the asynchronous conditions. (**d**) Questionnaire results regarding 3PP-hands. The ratings regarding body ownership (Q2), and agency (Q6) were significantly higher in the synchronous condition than in the asynchronous condition, but not in the case of 3PP-subjective tactile location (Q4). (**e**) SCR results regarding 3PP-hands. SCR was measured when the 3PP-hands were threatened with a knife. There was a significant difference between the synchronous and the asynchronous conditions. Bold lines indicate the medians; upper and lower limits of the box plot indicate the 75^th^ and 25^th^ percentile. The error bars represent the whole range of the ratings of the statement. Significance levels: *p < 0.05; **p < 0.01; ***p < 0.001. Abbreviation: STL, subjective tactile location.

The procedure of Experiment 5 was the same as Experiment 3, except that the 1PP-hands (with red tags) seen via the HMD were the participant’s own hands, and the 3PP-hands (with blue tags) seen via the HMD were the experimenter’s hands. The four-hand illusion was not induced in this experiment, even though the medians of Q7 were significantly higher in the synchronous condition than in the asynchronous condition (Z = −2.248, p = 0.025; Fig. 8a). The medians of the red-tag statements showed no significant difference between the synchronous and the asynchronous conditions (Q1: Z = −0.439, p = 0.660; Q3: Z = −0.632, p = 0.527; Q5: Z = −0.378, p = 0.705; Fig. 8b), so as the SCR measured when the 1PP-hands were threatened (Z = −0.700, p = 0.484; Fig. 8c). For the blue-tag statements, the scores of body ownership and agency were significantly higher in the synchronous condition than in the asynchronous condition (Q2: Z = −3.137, p = 0.002; Q6: Z = −3.133, p = 0.002; Fig. 8d), but not in the case of subjective tactile location (Q4: Z = −0.722, p = 0.470; Fig. 8d). The SCR that reflected the threat to the 3PP-hands was significantly higher in the synchronous condition than in the asynchronous condition (Z = −2.112, p = 0.035; Fig. 8e). Control statement Q8 showed no difference between the synchronous and the asynchronous conditions (Z = −1.000, p = 0.317).

#### Experiment 6

**Figure 9:**
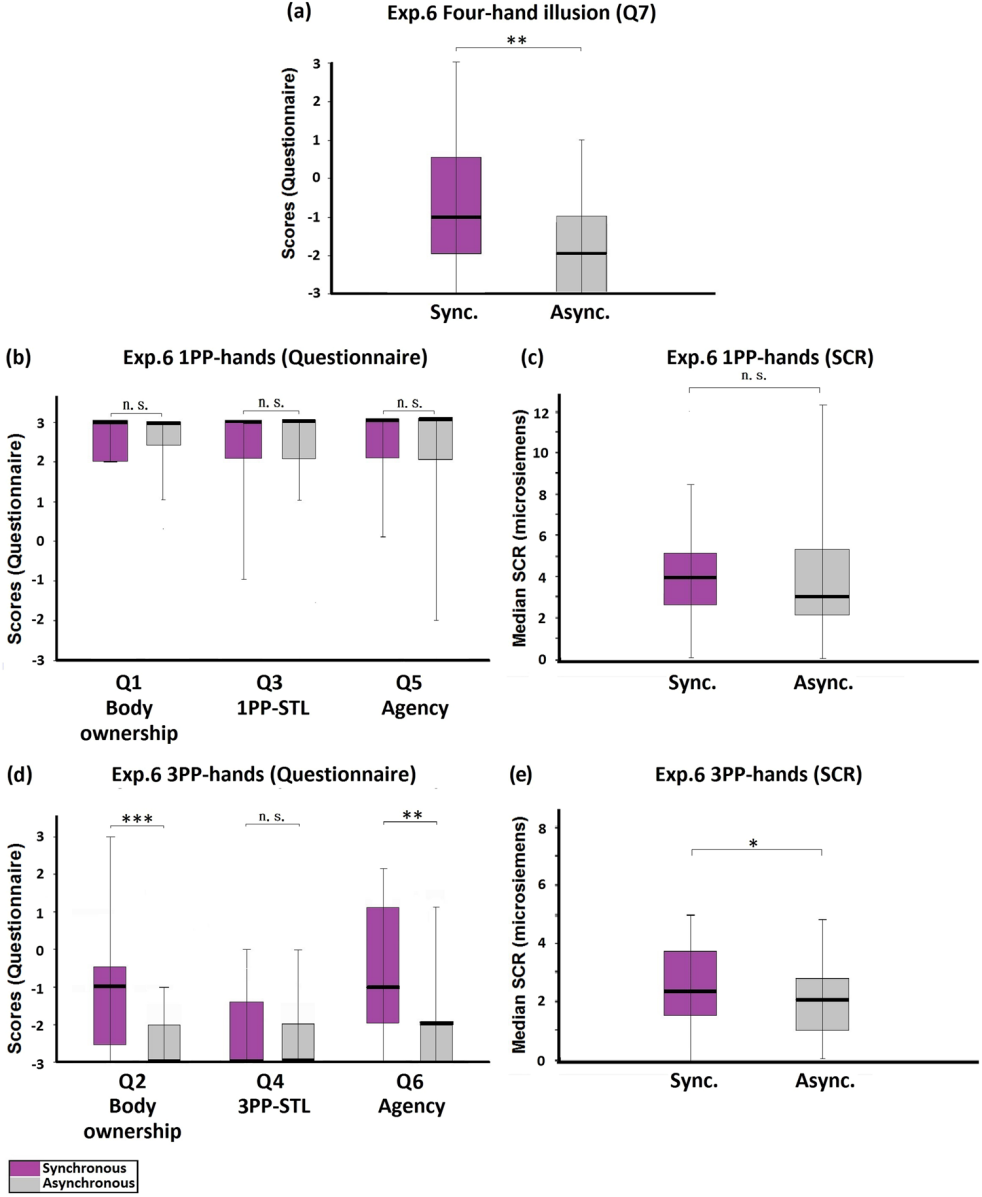
Experiment 6: Control active four-hand condition. (**a**) Questionnaire results regarding the four-hand illusion (Q7). Although a significant difference was observed between the synchronous and the asynchronous conditions, the medians in both conditions were negative. (**b**) Questionnaire results regarding 1PP-hands. There were no differences between the ratings regarding body ownership (Q1), 1PP-subjective tactile location (Q3), and agency (Q5). All the medians were very high. (**c**) SCR results regarding 1PP-hands. SCR was measured when the 1PP-hands were threatened with a knife. No significant difference was observed between the synchronous and the asynchronous conditions. (**d**) Questionnaire results regarding 3PP-hands. The ratings regarding body ownership (Q2), and agency (Q6) were significantly higher in the synchronous condition than in the asynchronous condition, while the ratings regarding 3PP-subjective tactile location (Q4) showed no significant difference. (**e**) SCR results regarding 3PP-hands. SCR was measured when the 3PP-hands were threatened with a knife. A significant difference existed between the synchronous and the asynchronous conditions. Bold lines indicate the medians; upper and lower limits of the box plot indicate the 75^th^ and 25^th^ percentile. The error bars represent the whole range of the ratings of the statement. Significance levels: *p < 0.05; **p < 0.01; ***p < 0.001. Abbreviation: STL, subjective tactile location.

The procedure of Experiment 6 was the same as Experiment 5, except that the brushing was asynchronous in both the synchronous and the asynchronous conditions. The four-hand illusion was not induced in this experiment, even though the medians of Q7 were significantly higher in the synchronous condition than in the asynchronous condition (Z = −2.782, p = 0.005; Fig. 9a). The medians of the red-tag statements showed no significant difference between the synchronous and the asynchronous conditions (Q1: Z = −1.134, p = 0.257; Q3: Z = −1.027, p = 0.305; Q5: Z = −0.359, p = 0.719; Fig. 9b), so as the SCR measured when the 1PP-hands were threatened (Z = −1.453, p = 0.146; Fig. 9c). For the blue-tag statements, the scores of body ownership and agency were significantly higher in the synchronous condition than in the asynchronous condition (Q2: Z = −3.482, p < 0.001; Q6: Z = −2.803, p = 0.005; Fig. 9d), but not in the case of subjective tactile location (Q4: Z = −0.333, p = 0.739; Fig. 9d). The SCR that reflected the threat to the 3PP-hands was significantly higher in the synchronous condition than in the asynchronous condition (Z = −2.085, p = 0.037; Fig. 9e). Control statement Q8 showed no difference between the synchronous and the asynchronous conditions (Z = 0.000, p = 1.000).

## Discussion

In this study, we combined 1PP and 3PP, and introduced both body agency and visual agency into the set-ups. These maneuvers provided useful ways to extend the flexibility of the sense of body ownership, and revealed the relevant constraints. As expected, the four-hand illusion was not induced in Experiment 1. But we found something interesting in this passive four-hand condition: the participants felt as if the tactile sensations were located in the 1PP-hands rather than in the 3PP-hands. Although the subjects felt that the 3PP-hands were their own, they did not feel the tactile sensations as located there. This suggests that the sense of 3PP-hand ownership and 3PP-subjective tactile location were dissociable. In Experiment 2, the active four-hand condition without touch, the participants did not feel either ownership of the 1PP-hands or the four-hand illusion, suggesting that finger movement alone was not sufficient for the key illusion to occur.

As reported above, the four-hand illusion was induced in the synchronous condition of Experiment 3. This was additionally supported by various analyses. First, different comparisons respectively showed that both the sense of ownership of the 1PP-hands and the sense of ownership of the 3PP-hands were induced in this active four-hand condition. Second, compared with Experiments 1 and 2, the sense of body ownership and the sense of agency of the 1PP-hands were significantly stronger in Experiment 3. Regarding the 3PP-hands, the sense of body ownership was slightly stronger (though not significantly) in Experiment 3 than in Experiment 1, and the sense of agency was roughly the same. Third, the score of four-hand illusion (Q7) in the synchronous condition of Experiment 3 was significantly higher than both the asynchronous condition of the same experiment and the synchronous conditions of both Experiments 1 and 2. Together these results suggest that the four-hand illusion was successfully induced in Experiment 3. The synchronous finger tapping and synchronous tactile stimulations jointly contributed to inducing the four-hand illusion.

Although the synchronous finger movement introduced in Experiment 3 played a crucial role in the four-hand illusion, the three other active four-hand experiments (Experiments 4~6) showed that synchronous movement alone was not sufficient for this novel illusion. The results of Experiment 4 indicate that the four-hand illusion was abolished when the brushing was asynchronous. Regarding the ownership of the 3PP-hands, it would require not only synchronous movement but also synchronous touch and a strong visual form congruence in order to overcome postural incongruence. Experiments 5 and 6 show that, if the 1PP-hands were the participant’s own hands, the visual form congruence of the 1PP-hands would be stronger than that of the 3PP-hands, such that the ownership of the 3PP-hands would be hindered. This would in turn obstruct the four-hand illusion whether the brushing was synchronous (Experiment 5) or asynchronous (Experiment 6). Given our set-ups, in order for the four-hand illusion to occur, the experimenter’s hands should be seen from the adopted 1PP, and the participant’s own hands from the adopted 3PP, and all four hands receive synchronous tactile stimulations. Only then the synchronous finger movement, more precisely, the synchronous body agency and visual agency, could substantially enhance the flexibility of the sense of body ownership. Below we discuss the implications of our findings, compare our experiments with other studies and respond to possible objections.

### Agency, Ownership and Postural incongruence

Some RHI studies appear to be at odds with our view that, under suitable manipulations, agency can strengthen and enhance the flexibility of the sense of body ownership. Tsakiris *et al*. (2006) reported that “In the active movement condition, the perceptual shifts were lower overall than the tactile and passive conditions”^19^ (p. 430). Kalckert and Ehrsson (2014) observed that “participants experienced the rubber hand illusion irrespective of whether it was elicited by active or passive index finger movements or by brushstrokes applied to the fingers. The strength of the illusion, as rated in the questionnaires, was not significantly different”^20^ (p. 127). Walsh *et al*. (2011) also reported that “active congruent movements (i.e. voluntary movements) produced an illusion that was the same or weaker than that produced by passive congruent movements”^21^ (p. 3019). We have no dispute with the results of these previous studies. Overall, the literature seems to suggest that whether agency might strengthen or diminish the sense of ownership depends on experimental designs. Here we will just point out the methodological difference between our study and these previous studies, and defend a modest position. One common feature among the above studies was that the key manipulations (tactile stimulations, active movement and passive movement) were implemented one at a time in *separate* experiments. In contrast, in the synchronous condition of our Experiment 3, the participant experienced both tactile stimulations and active movement at the same time, and saw all four hands acting with the same pattern. Implementing these manipulations *jointly* constitutes the main difference between our experiment and those previous studies. We are happy to limit our view to the specific set-ups of the current study. We only suggest that that, due to the joint manipulations in our experiment, the sense of body ownership was strengthened and extended by body agency and visual agency.

Kalckert and Ehrsson (2012) showed that, when the seen fake hand was rotated 180°, “the feelings of ownership and agency can be dissociated”. In their Active Incongruent condition, postural incongruence “eliminates ownership but does not disrupt agency”^22^ (p. 9). Notice that the visual content in our Experiment 3 was very different from Kalckert and Ehrsson’s. The subjects in Kalckert and Ehrsson’s study saw a fake hand in a white glove with the unseen real hand wearing an identical glove, such that the subjects may or may not feel the fake hand to be theirs. In contrast, the participants in our Experiment 3 saw their own real hands from the 3PP, which exhibited a much higher degree of visual form congruence than the fake hand in Kalckert and Ehrsson’s study. We think that the strong visual form likeness and vividness of the real hands, synchronous tactile stimulations, plus synchronized visual agency and body agency, can explain why the sense of ownership and agency did not dissociate in our experiment.

Some RHI studies suggested that postural incongruence would hinder the illusion. For example, Tsakiris and Haggard (2005) suggested that “the RHI occurs only when the viewed rubber hand is in congruent posture with the participant’s unseen hand”^13^ (p. 83; please see Supplementary Information for discussion on Tsakiris’ model of body ownership). The fMRI study by Ehrsson *et al*. (2004) made the similar claim as well^23^. However, there is an important difference between these two studies and the current one. That is, while the above two studies used fake hands to induce ownership experience, what was seen from the 3PP in our Experiments 1, 2 and 3 were the subjects own real hands. Although the postures were incongruent, the 3PP-hands exhibited the highest degree of visual form congruence which fake hands lack. Given this methodological difference, we don’t think our results really contradict with those previous RHI studies. Also, we are not suggesting that postural incongruence can be easily overcome by any 3PP set-ups. In fact, we think it is hard to do so. We only suggest that it can be done in some particular contexts. Our data on the 3PP-hand ownership show that postural incongruence could be accommodated by strong visual form congruence (Experiment 1), and a clear sense of 3PP-hand ownership could be induced when visual agency and body agency came into play (Experiments 2 and 3).

### Objections and Responses

Finally, we consider two possible objections. First, some might think that seeing one’s body or body parts from the 3PP is similar to looking into a mirror, where one merely recognizes one’s image rather than undergoing a genuine bodily experience. For example, Petkova *et al*. (2011) have doubts about using a 3PP set-up in FBI studies, according to which viewing the virtual body from the 3PP could be just a visual self-recognition on a monitor “without necessarily experiencing a somatic illusion of ownership in the same way as in the rubber hand illusion or in the body-swap illusion”^24^ (p. 5). However, recent studies on the RHI and FBI have shown that, with synchronous visual-tactile sensations, the illusion induced while watching the mirror reflection of a rubber hand or a mannequin from the 3PP was more or less the same as that induced by directly viewing the rubber hand or a full-body from the 1PP^25–28^. Using the moving RHI paradigm^20,22^, Jenkinson and Preston, extended this effect and induced both body ownership and agency on the subject’s hand mirrored from the 3PP^25^. As mentioned above, in our previous study, a self-touching illusion was induced when the participant saw his/her body from the adopted 3PP, with the participant and the experimenter brushing each other’s hand synchronously^18^. Therefore, we believe that it is possible for 3PP set-ups to induce genuine bodily illusions.

Second, one might consider the possibility that during the synchronous condition of Experiment 3 the participants focused their visual attention sometimes on the 1PP-hands and sometimes on the 3PP-hands. That is, it might be possible that their sense of ownership of the 1PP-hands and of the 3PP-hands was not really generated at the same time. If so, the experiment only induced two separate experiences of owning different pairs of hands rather than a genuine four-hand illusion. Here is what we think: there are good reasons suggesting that the illusion generated by our experiment was a subjectively integrated experience. Both pairs of hands were spatially close to each other and visually salient via the HMD. It was not likely that the participants might watch one pair of hands tapping the index finger without noticing the other pair performing exactly the same movement. More importantly, as the questionnaire data reported above, the participants’ experiences of the 3PP-hands were influenced by their experiences of seeing the 1PP-hands. This suggests a tight link between the two pairs of hands. These considerations strongly support that a subjective illusion was created that involved seeing four active hands moving in unison. Hence, during the experiment there was at least a period of time in which the participants experienced a genuine four-hand illusion.

## Methods

### Materials and Participants

In this study, we used a stereo camera (Sony HDR-TD20V) and a head mounted display (HMD, Sony HMZ-T1). The skin conductance responses (SCR) were recorded with a Data Acquisition Unit Biopac MP35 (Goleta, USA). SCR has been regarded as a reliable method of measuring the sense of body ownership both in the RHI and full-body illusions studies (Ehrsson, 2009; Guterstam *et al*., 2011; Kalckert and Ehrsson, 2012; Armel and Ramachandran, 2003; Ehrsson *et al*., 2007; Ehrsson *et al*., 2008; Petkova and Ehrsson, 2009)^5,6,22,29–32^. Compared with asynchronous manipulations, the SCR results in synchronous conditions are inferred as showing the strength of the implicit sense of body ownership. If the participant feels as if the seen body or body-part is his/her own, a greater reaction would be caused when it is threatened. SCR can provide the physiological evidence of such a reaction because it is controlled by the automatic nervous system (ANS) and cannot be altered by participants’ reflective intentions. For questionnaires, we used a Likert scale from “strongly disagree” (−3) to “strongly agree” (+3). The questionnaire statements were randomly distributed and divided into four categories: body ownership, subjective tactile location, agency, and key illusion. The questionnaires were in Chinese when presented to the participants. Tables 1 and 2 present the English translations. “Subjective tactile location” concerns whether the tactile stimulations were felt as if they were located on the hands seen from the participants’ 1PP or on the hands seen from the 3PP. Since we distinguish between 1PP- and 3PP-subjective tactile locations, this category is related but not the same as “touch referral” in the RHI literature.

We used a screen-switch machine (ATEN, VM5808H, Taiwan) that can switch between the images taken by the stereo camera and other computer images. This allowed us to present the questionnaires on the HMD, and to better control the synchronous stimulations. All participants wore earphones and white noise was played during the experiment to mask any sounds made by the operation. Three separate experiments were performed in this study, each with two conditions. All six experiments were within-subject designed. Each experiment recruited a different group of 25 participants. Totally, we recruited 150 healthy volunteers (see Table 3 for the details of the subjects). All participants gave their written informed consent prior to the experiments. The persons whose hands shown in Fig. 1 gave their written informed consent as well. All experiments were conducted in accordance with the Declaration of Helsinki. This study was approved by the Research Ethics Committee of National Taiwan University (NTU-REC: 201501HS009).

**Table 3:** Overview of experiments.

Experiments	Description	Measurements	Participants (n)	Statistics
Exp. 1	Passive 4 hands Sync. touch *1PP: other hands*	Questionnaire & SCR	n = 25(♂8) M = 22.2 ± 2.3	Wilcoxon test (Sync. vs. Async.)
	Passive 4 hands Async. touch *1PP: other hands*			
Exp. 2	Sync. Active 4 hands No touch *1PP: other hands*	Questionnaire & SCR	n = 25(♂14) M = 23.2 ± 5.1	Wilcoxon test (Sync. vs. Async.)
	Async. Active 4 hands No touch *1PP: other hands*			
Exp. 3	Sync. Active 4 hands Sync. touch *1PP: other hands*	Questionnaire & SCR	n = 25(♂11) M = 22.7 ± 2.5	Wilcoxon test (Sync. vs. Async.)
	Async. Active 4 hands Sync. touch *1PP: other hands*			
Exp. 4	Sync. Active 4 hands Async. touch *1PP: other hands*	Questionnaire & SCR	**n = 25(**♂11) M = 25 ± 5.8	Wilcoxon test (Sync. vs. Async.)
	Async. Active 4 hands Async. touch *1PP: other hands*			
Exp. 5	Sync. Active 4 hands Sync. touch *1PP: own hands*	Questionnaire & SCR	n = 25(♂9) M = 22.0 ± 1.9	Wilcoxon test (Sync. vs. Async.)
	**Async. Active 4 hands** Sync. touch *1PP: own hands*			
Exp. 6	Sync. Active 4 hands Async. touch *1PP: own hands*	Questionnaire & SCR	n = 25(♂14) M = 22.3 ± 1.7	Wilcoxon test (Sync. vs. Async.)
	Async. Active 4 hands Async. touch *1PP: own hands*			

### Procedure

The participant wore an HMD connected with a stereo camera positioned right beside the experimenter. Both the participant and the experimenter placed their hands on a table. After the participant put on the HMD, a color tag was attached to the back of all four hands. We showed an irrelevant landscape image on the HMD to prevent the subject from seeing this tagging procedure. So the participant would not know whether his/her own hands were tagged as red or blue. After this was done, the screen was switched back to the images taken by the stereo camera. The stereo camera filmed all four hands of the experimenter and the participant. Through the HMD, the participant adopted the experimenter’s 1PP as if it was his/her own 1PP. We will call this *adopted 1PP*. Two assistants began to brush the experimenter’s and the participant’s hands either synchronously or asynchronously for 60 seconds. When the touching was synchronous, the strokes were done once per second on the back of the subject’s and the experimenter’s hands, starting a little below the wrist all the way through the middle finger. When the touching was asynchronous, the two hands within each pair were brushed at the same pace, but the brushing speed was different between the two pairs, with time differences ranging from about 0.4 to 0.6 seconds.

After the brushing period (and after the finger movement in Experiment 3, see below), we displayed a knife to the camera for one second to make sure that the subject could see it, and pretended to cut the experimenter’s or the subject’s hands from left to right. The order of “threatening” was randomized during all the experiments. The period of threat lasted about 4 seconds. Then we took another 5–6 seconds to measure the subject’s SCR. To do so, two single-use foam electrodes (Covidien, Inc., Mansfield, USA) were attached to the participant’s left palm. One was placed 2 cm below the thumb and the other 4 cm below the little finger. The sampling rate was 200 samples per second, and the Hardware Filter was set to 20 KHz Lowpass. Data were analyzed with the Biopac software AcqKnowledge v. 3.7.7. We followed the standard procedure (Dawson *et al*., 2007) and identified the amplitude of SCR as the difference between the maximal and minimal values of the responses within 5 seconds of the threat^33^. After each experiment, the participant orally answered the questionnaire (Table 1) presented on their HMD by saying the number of their rating. Those subjects who did not show any SCR amplitude were classified as non-responders, and were excluded from the analysis. If they did not follow the instructions to keep their hands still or did not tap their index fingers with a steady pattern, their data were also removed. Totally, we excluded the data of 14 participants including their SCR and questionnaires. To measure SCR, two single-use foam electrodes (Covidien, Inc., Mansfield, USA) were attached to the inner side of the participant’s left palm. One was placed 2 cm below the thumb and the other 4 cm below the little finger. The wires were carefully put under the participant’s arm. So, throughout the experiments, both the electrodes and the wires would not be seen by the participant via the HMD.

We adopted a relatively high standard when interpreting the questionnaire data, i.e., two criteria had to be met before we claimed that a genuine effect was observed: first, the comparison between the experimental group and the control group must reach a significant level; second, the median of the experimental group must at least be positive one (+1). The differences of SCR results between the synchronous and the asynchronous conditions were regarded as indicating the relevant differences in the questionnaires. There were five dependent variables: the measures of hand ownership, subjective tactile location, agency, key illusion (the four-hand illusion) and SCR. The questionnaire scores were ordinal and not normally distributed (Shapiro-Wilk test). The SCR data were not normally distributed, either. To compare between the synchronous and asynchronous conditions within each experiment, we used non-parametric Wilcoxon signed-rank tests to analyze both the questionnaires and the SCR data. For subjective ratings of the same statement, we chose Mann-Whitney *U* tests to compare between two different experiments. We also performed correlation analyses implemented in SPSS version 19.0 (SPSS Inc., Chicago, IL, USA) to ascertain the relations between two dependent variables in the same experiment. We took the same statement of questionnaire in Experiments 1, 2 and 3 and conducted comparisons (Kruskal-Wallis test) to analyze the data (Supplementary Information). Finally, we conducted Scheier-Ray-Hare-Tests (α = 0.05) and used Mann-Whitney *U* tests for post-hoc analysis (α = 0.017, Dunn-Šidák correction) to compare the questionnaire data between Experiments 3 and 4 and between Experiments 3 and 5 (Supplementary Information).

### Experiment 1: Passive four-hand condition

In Experiment 1, a blue tag was attached to the back of the subject’s two hands and a red tag on the back of those of the experimenter. Then the participant saw via the HMD an image of four hands: the experimenter’s hands (with red tags) were seen from the adopted 1PP, and the participant’s own hands (with blue tags) from the adopted 3PP (Fig. 1b). The subject and the experimenter kept their hands still while being brushed synchronously or asynchronously for one minute. After that, the experimenter’s hands and the subject’s hands were “threatened” in turn with a knife. There was a 10 second break between the first and the second threat, during which all four hands continued to be brushed. At the end of the experiment, a questionnaire was presented on the HMD. The whole procedure took about 110 seconds.

### Experiment 2: Active four-hand condition (without touch)

There was a training block before this experiment, where all participants practiced tapping their index fingers at a constant speed of about one tap per second. The experiment consisted of two stages. The procedure of the first stage was the same as the synchronous condition of Experiment 1, except that there was no brushing. All four hands remained still for 60 seconds. Then the second stage: both the participant and the experimenter were asked to close their hands into fists and then stretch out both of their index fingers to tap on the table about once per second (Fig. 1b). The screen was switched to an irrelevant landscape image just before the subject closed his/her hands into fists. After the subject began tapping for 4 seconds, the image was switched back to the camera. Then the tapping continued for another 10 seconds, followed by the same screen-switch maneuver. Right after that, the subject was instructed to stop tapping and open his/her fist, and then the screen was switched back to the camera. There was a break for 10 seconds, and then the same tapping cycle was repeated. The knife-threats commenced after these procedures were completed, i.e., the knife-threats began when both the finger tapping and the brushing came to stop. In the synchronous condition, the experimenter tapped his index fingers synchronously with respect to the participant’s, such that the participant saw all four hands acting in exactly the same pattern. In the asynchronous condition, the experimenter intentionally tapped his index fingers asynchronously with respect to the participant’s finger movements with time differences ranging from about 0.4 to 0.6 seconds. Notice that, since the 3PP-hands were the subject’s own hands, the 3PP-finger tapping seen via the HMD was still synchronous with respect to the subject’s finger movement felt via proprioception. Each condition was followed by knife-threats on the experimenter’s and the subject’s hands as well as the questionnaire on the HMD. The whole procedure took about 180 seconds.

### Experiment 3: Active four-hand condition (with touch)

Similar to Experiment 2, there was a training block before this experiment, where all participants practiced tapping their index fingers at a constant speed of about one tap per second. The experiment consisted of two stages as well. The procedure of the first stage was exactly the same as the synchronous condition of Experiment 1. All four hands were brushed synchronously for 60 seconds. Then the second stage: the participants were instructed to perform finger movement. The procedures of both the synchronous and the asynchronous conditions were the same as the second stage of Experiment 2, except that during the two tapping cycles, including the break in between, all four hands continued to be brushed synchronously. Like Experiment 2, in the asynchronous condition, since the 3PP-hands were the subject’s own hands the 3PP-finger tapping seen via the HMD was still synchronous with respect to the subject’s finger movement felt via proprioception. Each condition was followed by knife-threats on the experimenter’s and the subject’s hands as well as the questionnaire on the HMD. The whole procedure took about 180 seconds.

### Experiments 4~6: Control active four-hand conditions

We conducted three other active four-hand experiments as controls (Fig. 1c). Each consisted of two conditions (synchronous vs. asynchronous finger tapping) and recruited a different group of 25 subjects. The procedure of Experiment 4 was the same as Experiment 3, except that the brushing was asynchronous in both the synchronous and the asynchronous conditions. The procedure of Experiment 5 was the same as Experiment 3, except that the 1PP-hands (with red tags) seen via the HMD were the participant’s own hands, and the 3PP-hands (with blue tags) seen via the HMD were the experimenter’s hands. Finally, the procedure of Experiment 6 was the same as Experiment 5, except that the brushing was asynchronous in both the synchronous and the asynchronous conditions. A final note: there was a condition that we did not perform to compare with the synchronous condition of Experiment 3, i.e., the condition in which the finger tapping seen via the HMD was asynchronous with respect to participant’s body agency. The reason was that doing so would cause the brushing seen via the HMD to be asynchronous with respect to the tactile stimulations that the participants felt on their hands. The two factors (finger movement and touch) would be manipulated at the same time and confound with each other.

## Electronic supplementary material

Supplementary information

## References

[1] Botvinick M, Cohen J (1998). Rubber hands ‘feel’ touch that eyes see. Nature.

[2] Ehrsson HH (2007). The experimental induction of out-of-body experiences. Science.

[3] Lenggenhager B, Tadi T, Metzinger T, Blanke O (2007). Video ergo sum: manipulating bodily self-consciousness. Science.

[4] Blanke O, Metzinger T (2009). Full-body illusions and minimal phenomenal selfhood. Trends Cogn. Sci..

[5] Ehrsson HH (2009). How many arms make a pair? Perceptual illusion of having an additional limb. Perception.

[6] Guterstam A, Petkova VI, Ehrsson HH (2011). The illusion of owning a third arm. PLoS One.

[7] Newport R, Pearce R, Preston C (2010). Fake hands in action: embodiment and control of supernumerary limbs. Exp. Brain Res..

[8] Guterstam A, Gentile G, Ehrsson HH (2013). The Invisible Hand Illusion: Multisensory Integration leads to the Embodiment of a Discrete Volume of Empty Space. J. Cogn. Neurosci..

[9] Petkova VI, Ehrsson HH (2008). If I Were You: Perceptual Illusion of Body Swapping. PLoS One.

[10] Heydrich L (2013). Visual capture and the experience of having two bodies–Evidence from two different virtual reality techniques. Front. Psychol..

[11] Shimada S, Fukuda K, Hiraki K (2009). Rubber Hand Illusion under Delayed Visual Feedback. PLoS One.

[12] Pavani F, Zampini M (2007). The role of hand size in the fake-hand illusion paradigm. Perception.

[13] Tsakiris M, Haggard P (2005). The rubber hand illusion revisited: visuotactile integration and self-attribution. J. Exp. Psychol. Hum. Percept. Perform..

[14] Tsakiris M (2010). My body in the brain: A neurocognitive model of body-ownership. Neuropsychologia.

[15] Tsakiris M, Longo MR, Haggard P (2010). Having a body versus moving your body: Neural signatures of agency and body-ownership. Neuropsychologia.

[16] Lloyd DM (2007). Spatial limits on referred touch to an alien limb may reflect boundaries of visuo-tactile peripersonal space surrounding the hand. Brain Cogn..

[17] Costantini M, Haggard P (2007). The rubber hand illusion: Sensitivity and reference frame for body ownership. Conscious. Cogn..

[18] Liang C, Chang SY, Chen WY, Huang HC, Lee YT (2015). Body ownership and experiential ownership in the self-touching illusion. Front. Psychol..

[19] Tsakiris M, Prabhu G, Haggard P (2006). Having a body versus moving your body: how agency structures body-ownership. Conscious. Cogn..

[20] Kalckert A, Ehrsson HH (2014). The moving rubber hand illusion revisited: Comparing movements and visuotactile stimulation to induce illusory ownership. Conscious. Cogn..

[21] Walsh LD, Moseley GL, Taylor JL, Gandevia SC (2011). Proprioceptive signals contribute to the sense of body ownership. J. Physiol..

[22] Kalckert A, Ehrsson HH (2012). Moving a rubber hand that feels like your own: a dissociation of ownership and agency. Front. Hum. Neurosci..

[23] Ehrsson HH, Spence C, Passingham RE (2004). That’s my hand! Activity in premotor cortex reflects feeling of ownership of a limb. Science.

[24] Petkova VI, Khoshnevis M, Ehrsson HH (2011). The perspective matters! Multisensory integration in ego-centric reference frames determines full-body ownership. Front. Psychol..

[25] Jenkinson PM, Preston C (2015). New reflections on agency and body ownership: The moving rubber hand illusion in the mirror. Conscious. Cogn..

[26] Bertamini M, Berselli N, Bode C, Lawson R, Wong LT (2011). The rubber hand illusion in a mirror. Conscious. Cogn..

[27] Kontaris I, Downing PE (2011). Reflections on the hand: The use of a mirror highlights the contributions of interpreted and retinotopic representations in the rubber-hand illusion. Perception.

[28] Preston C, Kuper-Smith BJ, Ehrsson HH (2015). Owning the body in the mirror: The effect of visual perspective and mirror view on the full-body illusion. Scientific Reports.

[29] Armel KC, Ramachandran VS (2003). Projecting sensations to external objects: evidence from skin conductance response. Proc. Biol. Sci..

[30] Ehrsson HH, Weich K, Weiskopf N, Dolan RJ, Passingham RE (2007). Threatening a rubber hand that you feel is yours elicits a cortical anxiety response. Proc. Natl. Acad. Sci. USA.

[31] Ehrsson HH (2008). Upper limb amputees can be induced to experience a rubber hand as their own. Brain.

[32] Petkova VI, Ehrsson HH (2009). When right feels left: referral of touch and ownership between the hands. PLoS One.

[33] Dawson, M. E., Schell, A. M. & Filion, D. L. The electrodermal system in *Handbook of* Psychophysiology (eds Cacioppo, J. T., Tassinary, L. G. & Berntson, G.) Ch. 8, 200–223 (Cambridge University Press, 2006).

