# Supplementary material for: Body ownership and the four-hand illusion
Source: Sci Rep. 2018 Feb 1;8:2153. doi: 10.1038/s41598-018-19662-x (PMC5794744; doi:10.1038/s41598-018-19662-x)
Supplement: Supplementary file 1 — Supplementary information [file 41598_2018_19662_MOESM1_ESM.pdf]

Body ownership and the four-hand illusion

Wen-Yeo Chen[1], Hsu-Chia Huang [1], Yen-Tung Lee[2], Caleb Liang[1, 2]

[1] Graduate Institute of Brain and Mind Sciences, National Taiwan University, Taiwan

[2] Department of Philosophy, National Taiwan University, Taiwan

Supplementary information

The supplementary information consists of four parts. In the first part, we report an additional experiment related to our study. In the second part, we report some secondary experimental results. In the third and fourth parts, we continue to discuss the implications of our findings and compare with other studies.

I. Passive two-hand condition

In Passive two-hand condition (Suppl. figure 1), a red tag was attached to the back of the participant's and the experimenter's hands. Then, via the HMD, the subject saw the experimenter's two hands (with red tags) from the adopted 1PP. The experimenter's two hands were brushed synchronously or asynchronously with respect to the participant's own unseen hand, followed by a knife-threat and a questionnaire presented on the HMD. The whole procedure took about 90 seconds. Since in this set-up the subject saw only two hands, the questionnaire used in this experiment included only Q1, Q3, Q5, Q7 and Q8 in Table 1 of the main text. Statements Q2, Q4, Q6 did not apply here, and Q7 was rephrased to adjust for this two-hand setting. We wanted to see whether this setting would generate a variant of the RHI with a pair of hands, and the results of this experiment would be compared with the data collected in other experiments.

Suppl. figure 1. Image of the passive two-hand condition.

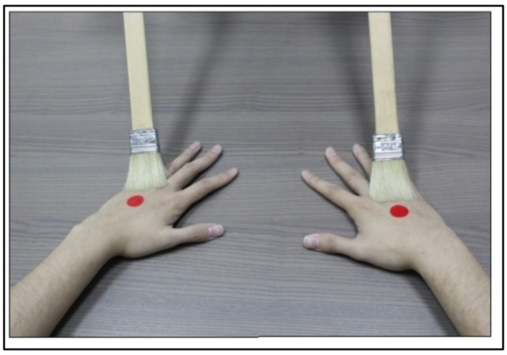

Suppl. figure 1. Image of the passive two-hand condition.
The participant saw a pair of hands via the HMD: the experimenter's hands from the adopted 1PP with red tags. The participant passively received tactile stimulations.

Suppl. table 1. Median Values and interquartile ranges (IQRs) of questionnaire statements and SCR in the Passive two-hand condition.

questionnaires	Experiment 1 Median (IQR)	
	Sync.	Async.
1. It felt as if the hands with red tags were mine.	2 (0~2)	-1 (-2~1)
3. The touches that I felt were located on the hands with red tags.	1 (-0.5~2)	-1 (-2~0)
5. It felt as if I could control the hands with red tags.	1 (-0.5~1)	-1 (-2~0.5)
7. At a certain point, I felt as if the two hands that I saw were both mine.	2 (-1~3)	-1 (-2~1)
8. I felt that my hands were brushed.	3 (3~3)	3 (3~3)
SCR on 1PP-hands	3.2 (1.5~6.8)	1.7 (0.7~3.2)

We found that the synchronous condition exhibited stronger agreement than the asynchronous condition in statements regarding body ownership (Q1, $Z = -3.145$, $p = 0.002$), subjective tactile location (Q3, $Z = -3.721$, $p < 0.001$), agency (Q5, $Z = -3.131$, $p = 0.002$), and two-hand illusion (Q7, $Z = -2.994$, $p = 0.003$) (Suppl. figure 2a). The control statement Q8 showed no such difference ($Z = -0.577$, $p = 0.564$). When the *1PP-hands* (the hands seen from the adopted 1PP via the HMD) were threatened by a knife, the SCR measured in the synchronous condition was also higher than that in the asynchronous condition ($Z = -3.229$, $p = 0.001$) (Suppl. figure 2b). These results suggest that the Passive two-hand condition successfully induced a version of RHI on a pair of hands, i.e., the participants felt as if the hands in front of them were theirs, they felt as if the tactile sensations were located on the 1PP-hands, and they felt as if they could control them.

Suppl. figure 2. Results of Passive two-hand condition.

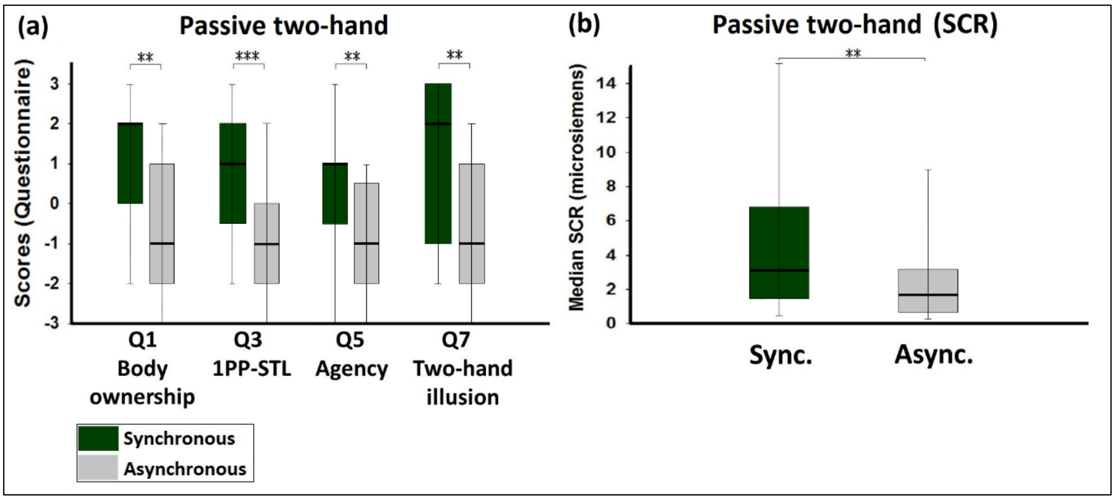

Suppl. figure 2. Results of Passive two-hand condition.

(a) Questionnaire results. The subjective ratings on the 1PP-hands seen via the HMD regarding body ownership (Q1), 1PP-subjective tactile location (Q3), agency (Q5), and the two-hand illusion (Q7) were significantly higher in the synchronous than in the asynchronous condition. **(b) SCR results.** SCR was measured when the experimenter's hands seen via the HMD were threatened by a knife. There was a significant difference between the synchronous and the asynchronous conditions. Bold lines indicate the medians; upper and lower limits of the box plot indicate the 75th and 25th percentile. The error bars represent the whole range of the ratings of the statement. Significance levels: *p<0.05; **p<0.01; ***p<0.001. Abbreviation: STL, subjective tactile location.

Although all subjects kept their hands static, in the synchronous condition many of them (Q5, 14 out of 25 subjects, 56%) still felt a weak sense of agency, and the relation between body ownership (Q1) and agency (Q5) showed a high positive correlation (Pearson's correlation = 0.758, $p < 0.001$, two tailed, with $\alpha = 0.01$). This result was consistent with a recent study by Kalckert and Ehrsson (2014). They reported that in the synchronous passive conditions, if subjects felt that the rubber hand was theirs, then they would also feel that they could control it to a certain extent[1] (pp. 122–123).

We compared the induced sense of body ownership regarding 1PP-hands (Q1) in the synchronous conditions of the Passive two-hand condition, Experiment 1 and Experiment 3 by conducting non-parametrical Kruskal-Wallis test (for multiple comparisons). The result revealed a significant difference ($H = 8.266$, $p = 0.016$, $\alpha = 0.05$). Further analyses (Mann-Whitney *U* test, $\alpha = 0.017$, Dunn-Šidák correction) showed that Q1 in the Passive two-hand condition was significantly higher than that in

Experiment 1 (Z = -2.476, p = 0.013). Q1 in Experiment 3 was significantly higher than that in Experiment 2 as well (Z = -2.478, p = 0.013). The comparison of Q1 between the Passive two-hand condition and Experiment 3 showed no significant difference (Z = -0.288, p = 0.773; Suppl. figure 3).

Suppl. figure 3. Multiple comparisons of Q1 in the synchronous conditions of the Passive two-hand condition, Experiment 1 and Experiment 3.

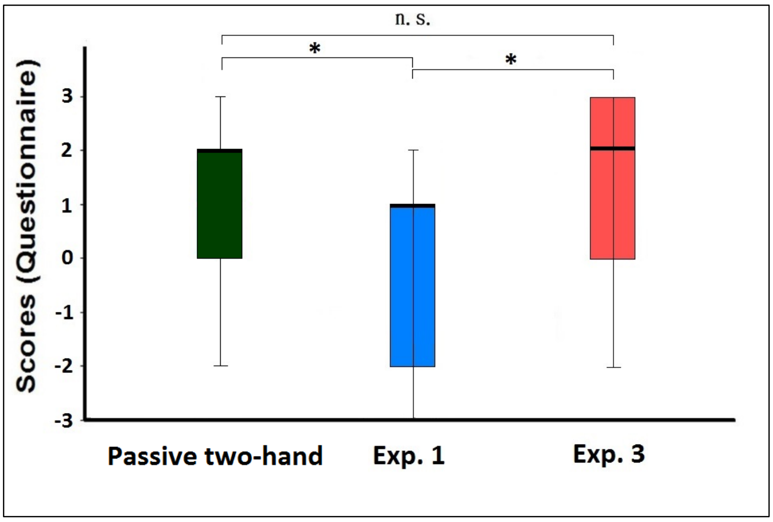

Suppl. figure 3. Multiple comparisons of Q1 in the synchronous conditions of the Passive two-hand condition, Experiment 1 and Experiment 3. Body ownership regarding 1PP-hands (Q1) in both the Passive two-hand condition and Experiment 3 were significantly higher than that in Experiment 1. The comparison of Q1 between the Passive two-hand condition and Experiment 3 showed no significant difference. Bold lines indicate the medians; upper and lower limits of the box plot indicate the 75th and 25th percentile. The error bars represent the whole range of the ratings of the statement. Significance levels: $*p<0.05$; $**p<0.01$; $***p<0.001$. Abbreviation: n. s., not significant; 1PP, first-person perspective.

II. Other experimental results

We compared the scores between the 1PP-hands and the 3PP-hands in the synchronous condition of **Experiment 2**. We found no significant differences between Q1 (ownership of 1PP-hands) and Q2 (ownership of 3PP-hands) (Z = -0.700, p = 0.865) and between Q3 (1PP-subjective tactile location) and Q4 (3PP-subjective tactile location) (Z = -1.210 p = 0.210). But there was a significant difference between Q5 (agency of 1PP-hands) and Q6 (agency of 3PP-hands) (Z = -3.028, p = 0.002, Suppl. figure 4a).

When comparing the scores between the 1PP-hands and the 3PP-hands in the

synchronous condition of **Experiment 3**, we found no significant differences between Q1 (ownership of 1PP-hands) and Q2 (ownership of 3PP-hands) ($Z = -0.190$, $p = 0.985$), between Q3 (1PP-subjective tactile location) and Q4 (3PP-subjective tactile location) ($Z = -1.827$, $p = 0.068$), and between Q5 (agency of 1PP-hands) and Q6 (agency of 3PP-hands) ($Z = -0.560$, $p = 0.955$, Suppl. figure 4b). This indicates that, in the synchronous condition of Experiment 3, the induced subjective experiences of the 1PP-hands and the 3PP-hands were roughly the same.

We compared the scores between the 1PP-hands and the 3PP-hands in the synchronous condition of **Experiment 4**, and found significant differences between Q1 (ownership of the 1PP-hands) and Q2 (ownership of the 3PP-hands) ($Z = -4.184$, $p < 0.001$), between Q3 (1PP-subjective tactile location) and Q4 (3PP-subjective tactile location) ($Z = -4.354$, $p < 0.001$), and between Q5 (agency of the 1PP-hands) and Q6 (agency of the 3PP-hands) ($Z = -3.915$, $p < 0.001$, Suppl. figure 4c). These data indicate that, in the synchronous condition of Experiment 4, the induced subjective experiences of the 1PP-hands and of the 3PP-hands were rather different.

When comparing the scores between the 1PP-hands and the 3PP-hands in the synchronous condition of **Experiment 5**, we found significant differences between Q1 (ownership of the 1PP-hands) and Q2 (ownership of the 3PP-hands) ($Z = -4.305$, $p < 0.001$), between Q3 (1PP-subjective tactile location) and Q4 (3PP-subjective tactile location) ($Z = -4.455$, $p < 0.001$), and between Q5 (agency of the 1PP-hands) and Q6 (agency of the 3PP-hands) ($Z = -4.392$, $p < 0.001$, Suppl. figure 4d). These data indicate that, in the synchronous condition of Experiment 5, the induced subjective experiences of the 1PP-hands and the 3PP-hands were significantly different.

When comparing the scores between the 1PP-hands and the 3PP-hands in the synchronous condition of **Experiment 6**, we also found significant differences between Q1 (ownership of the 1PP-hands) and Q2 (ownership of the 3PP-hands) ($Z = -4.311$, $p < 0.001$), between Q3 (1PP-subjective tactile location) and Q4 (3PP-subjective tactile location) ($Z = -4.399$, $p < 0.001$), and between Q5 (agency of the 1PP-hands) and Q6 (agency of the 3PP-hands) ($Z = -4.229$, $p < 0.001$, Suppl. figure 4e). These data indicate, in the synchronous condition of Experiment 6, the induced subjective experiences of the 1PP-hands and the 3PP-hands were very different.

Suppl. figure 4. 1PP-hands vs. 3PP-hands within synchronous conditions

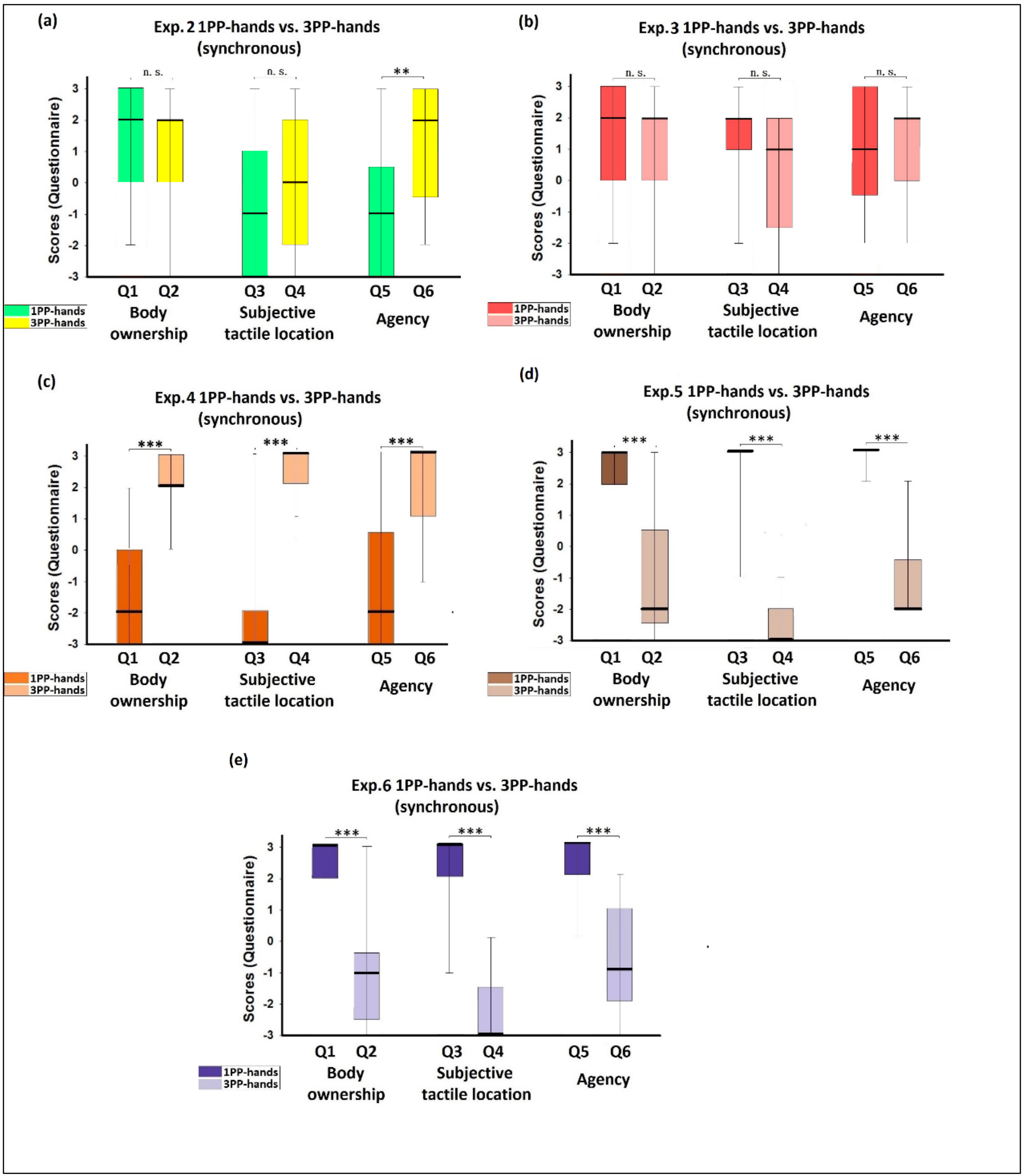

Suppl. figure 4. Comparisons between 1PP-hands and 3PP-hands within synchronous conditions. (a) Experiment 2. Neither the ratings of body ownership nor that of subjective tactile location showed significant differences. The rating of agency of the 1PP-hands was significantly lower than that of the 3PP-hands. **(b) Experiment 3.** There were no significant differences between the ratings of 1PP-hands and 3PP-hands regarding body ownership, subjective tactile location, and agency. **(c) Experiment 4.** The ratings of the 3PP-hands were significantly higher than that of the 1PP-hands. **(d) Experiment 5.** The ratings of the 1PP-hands were significantly higher than those of the 3PP-hands. **(e) Experiment 6.** The ratings of the 1PP-hands were significantly higher than those of the 3PP-hands. Bold lines indicate the medians; upper

and lower limits of the box plot indicate the 75th and 25th percentile. The error bars represent the whole range of the ratings of the statement. Significance levels: *p<0.05; **p<0.01; ***p<0.001. Abbreviation: n. s., not significant.

We totally conducted four active four-hand experiments. The two factors were "touch" (synchronous vs. asynchronous) and "perspective" (1PP-hands: experimenter's hands vs. participant's own hands). Regarding the factor "touch", the touches in Experiments 3 and 5 were all synchronous. In contrast, the touches in Experiments 4 and 6 were all asynchronous. The factor "perspective" was operationalized between Experiments 3 and 5, and between Experiments 4 and 6. In Experiments 3 and 4, the 1PP-hands were the experimenter's hands, and the 3PP-hands were the participant's own hands. In Experiments 5 and 6, the 1PP-hands were the participant's own hands, and the 3PP-hands were the experimenter's hands. Overall, this was a two-by-two between-design (Suppl. table 2). Notice that, the factor "visual form" (own vs. other hands) in fact represents the same meaning as the factor "perspective" in our setting. Due to the fact that there were always two pairs of hands on the table and one of which was always the participant's, the factor "perspective" determined whose hands would be viewed from 1PP and from 3PP respectively. Hence, it was not important to separate the factor "visual form" from "perspective" in our experiments.

Suppl. table 2.

Perspective / Touch	1PP: experimenter's hands	1PP: participant's own hands
Synchronous.	Exp. 3	Exp. 5
Asynchronous.	Exp. 4	Exp. 6

We then performed Scheier-Ray-Hare-Test (α = 0.05) and used Mann-Whitney *U* tests for post-hoc analyses (α = 0.017, Dunn-Šidák correction) to compare the questionnaire data between Experiments 3 and 4 (Suppl. figure 5) and between Experiments 3 and 5 (Suppl. figure 6). The results of Scheier-Ray-Hare-Test for each questionnaire statement were presented in Supplementary table 3 below. In Q1, Q4, and Q7, main effects on both factors were observed as well as interaction effect. In Q3 and Q5, there were main effects but no interaction effect. In Q2 and Q6, only the factor "perspective" was statistically significant.

Suppl. table 3.

Scheirer-Ray-Hare-Test, α = 0.05 (sync. conditions of Exp.3, 4, 5 and 6)					Post-hoc, α = 0.017	
					Exp.3 vs. Exp.4	Exp.3 vs. Exp.5
1PP-hands	Q1	Perspective	$H_{(1,96)} = 47.223$	$p < 0.001$	Z = -4.303 p < 0.001	Z = -3.763 p < 0.001
		Touch	$H_{(1,96)} = 8.496$	$p = 0.004$		
		Interaction	$H_{(1,96)} = 4.949$	$p = 0.026$		
	Q3	Perspective	$H_{(1,96)} = 44.794$	$p < 0.001$	Z = -5.092 p < 0.001	Z = -4.506 p < 0.001
		Touch	$H_{(1,96)} = 12.641$	$p < 0.001$		
		Interaction	$H_{(1,96)} = 2.821$	$p = 0.093$		
	Q5	Perspective	$H_{(1,96)} = 42.242$	$p < 0.001$	Z = -4.132 p < 0.001	Z = -4.144 p < 0.001
		Touch	$H_{(1,96)} = 11.649$	$p = 0.001$		
		Interaction	$H_{(1,96)} = 2.081$	$p = 0.149$		
3PP-hands	Q2	Perspective	$H_{(1,96)} = 46.758$	$p < 0.001$	Z = -3.156 p = 0.002	Z = -3.983 p < 0.001
		Touch	$H_{(1,96)} = 2.361$	$p = 0.124$		
		Interaction	$H_{(1,96)} = 2.492$	$p = 0.114$		
	Q4	Perspective	$H_{(1,96)} = 62.487$	$p < 0.001$	Z = -5.226 p < 0.001	Z = -5.045 p < 0.001
		Touch	$H_{(1,96)} = 5.236$	$p = 0.022$		
		Interaction	$H_{(1,96)} = 5.156$	$p = 0.023$		
	Q6	Perspective	$H_{(1,96)} = 42.392$	$p < 0.001$	Z = -2.509 p = 0.012	Z = -4.031 p < 0.001
		Touch	$H_{(1,96)} = 2.498$	$p = 0.114$		
		Interaction	$H_{(1,96)} = 1.193$	$p = 0.275$		
4-hand illusion	Q7	Perspective	$H_{(1,96)} = 6.754$	$p = 0.009$	Z = -3.700 p < 0.001	Z = -4.085 p < 0.001
		Touch	$H_{(1,96)} = 5.410$	$p = 0.020$		
		Interaction	$H_{(1,96)} = 9.647$	$p = 0.002$		

For the post-hoc analyses, we first compared between the synchronous conditions of Experiments 3 and 4. Regarding the four-hand illusion (Q7), the rating in Experiment 3 was significantly higher than that in Experiment 4 ($Z = -3.700$, $p < 0.001$; Suppl. figure 5a). The ratings regarding the 1PP-hands in Experiment 3 were significantly higher than those in Experiment 4 (Q1: $Z = -4.303$, $p < 0.001$; Q3: $Z = -5.092$, $p < 0.001$; Q5: $Z = -4.132$, $p < 0.001$; Suppl. figure 5b). In Experiment 3, the responses regarding the 3PP-hands were significantly lower than those in Experiment 4. (Q2: $Z = -3.156$, $p = 0.002$; Q4: $Z = -5.226$, $p < 0.001$; Q6: $Z = -2.509$, $p = 0.012$; Suppl. figure 5c). These data indicate that the subjective experiences induced in the synchronous condition of

Experiment 3 were very different from those in Experiment 4.

Then we compared between the synchronous conditions of Experiments 3 and 5. The rating of the four-hand illusion (Q7) in Experiment 3 was significantly higher than that in Experiment 5 ($Z = -4.085$, $p < 0.001$; Suppl. figure 6a). The ratings regarding the 1PP-hands in Experiment 5 were significantly higher than those in Experiment 3 (Q1: $Z = -3.763$, $p < 0.001$; Q3: $Z = -4.506$, $p < 0.001$; Q5: $Z = -4.144$, $p < 0.001$; Suppl. figure 6b). In Experiment 3, the responses regarding the 3PP-hands were significantly higher than those in Experiment 5 (Q2: $Z = -3.983$, $p < 0.001$; Q4: $Z = -5.045$, $p < 0.001$; Q6: $Z = -4.031$, $p < 0.001$; Suppl. figure 6c). These data suggest that the subjective experiences induced in the synchronous conditions of Experiments 3 and 5 were rather different.

Suppl. figure 5. Comparisons between Experiment 3 and Experiment 4.

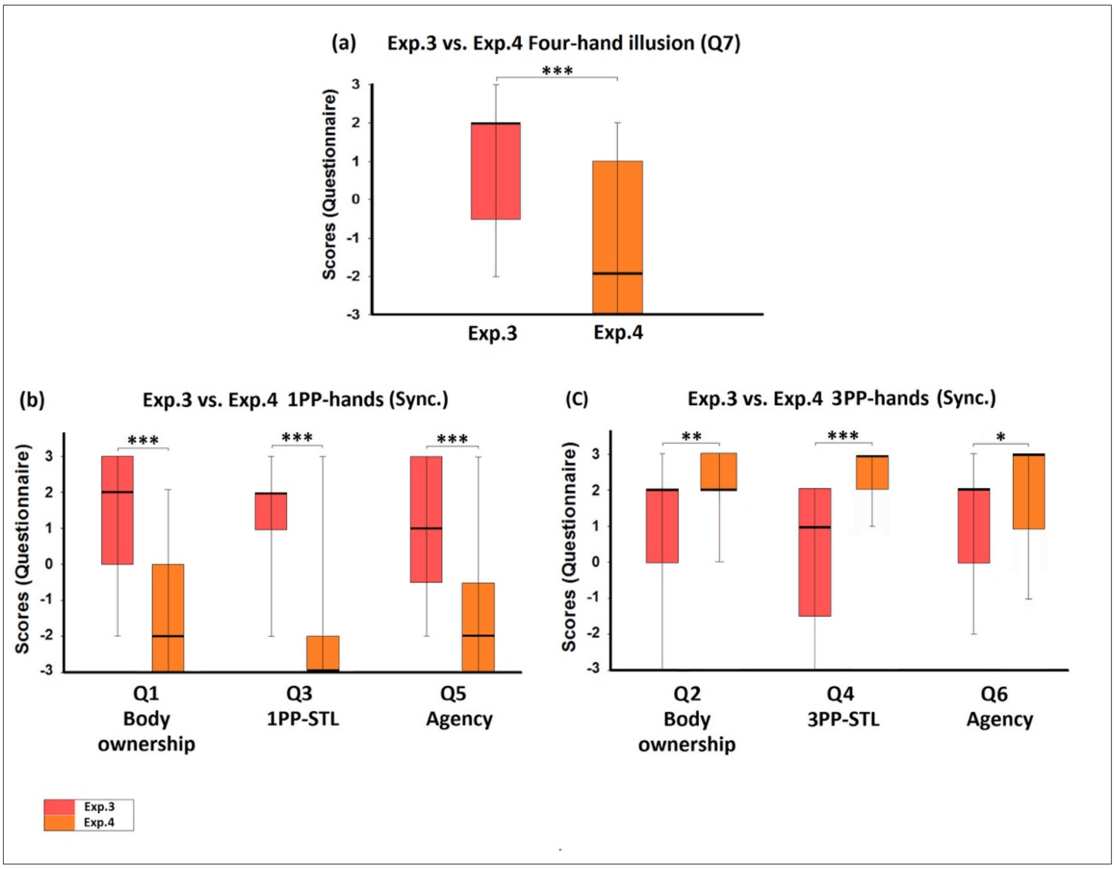

Suppl. figure 5. Comparisons between Experiment 3 and Experiment 4. (a) Comparison regarding the four-hand illusion. The ratings of the four-hand illusion (Q7) in Experiment 3 were significantly higher than that in Experiment 4. **(b) Comparison regarding 1PP-hands.** The ratings of the 1PP-hands in Experiment 3 regarding body ownership (Q1), 1PP-subjective tactile location (Q3) and agency (Q5)

were significantly higher than that in Experiment 4. **(c) Comparison regarding 3PP-hands.** The ratings of 3PP-hands in Experiment 3 regarding body ownership (Q2), 3PP-subjective tactile location (Q4) and Agency (Q6) were significantly lower than that in Experiment 4. Bold lines indicate the medians; upper and lower limits of the box plot indicate the 75th and 25th percentile. The error bars represent the whole range of the ratings of the statement. Significance levels: *p<0.05; **p<0.01; ***p<0.001. Abbreviation: STL, subjective tactile location.

Suppl. figure 6. Comparisons between Experiment 3 and Experiment 5.

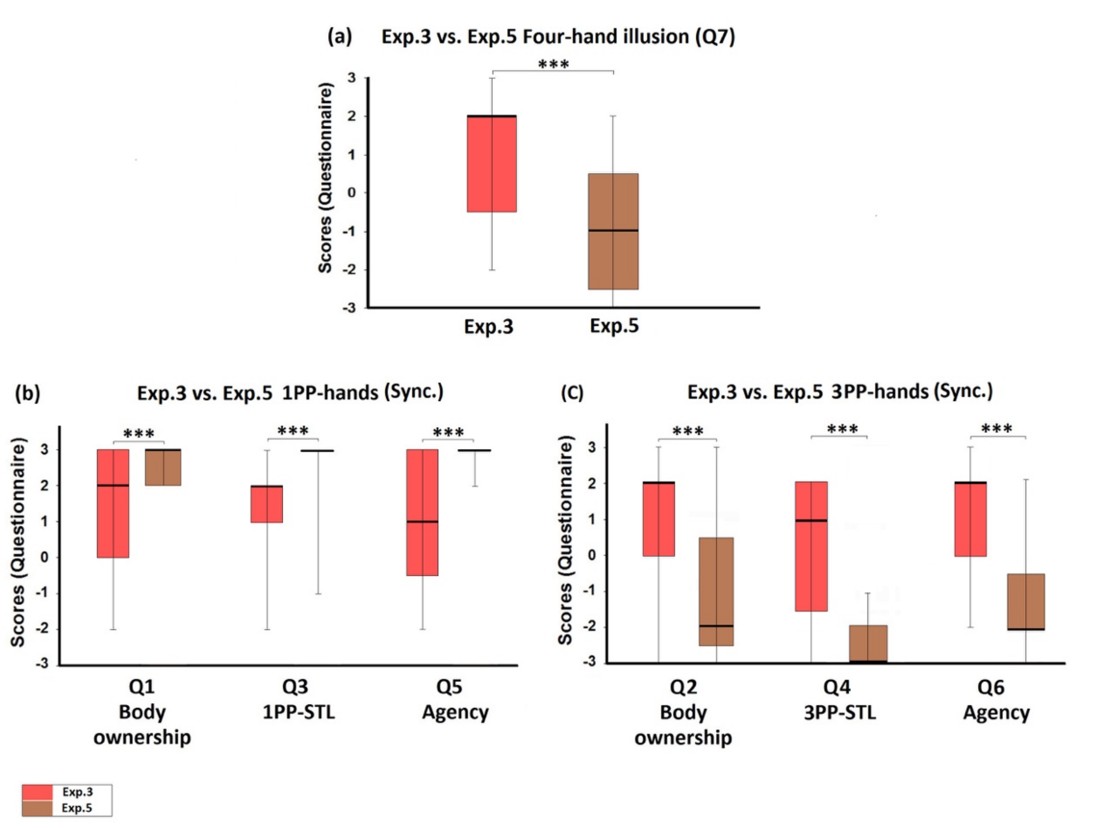

Suppl. figure 6. Comparisons between Experiment 3 and Experiment 5. (a) Comparison regarding the four-hand illusion. The ratings of the four-hand illusion (Q7) in Experiment 3 were significantly higher than that in Experiment 5. **(b) Comparison regarding 1PP-hands.** The ratings of 1PP-hands in Experiment 5 regarding body ownership (Q1), 1PP-subjective tactile location (Q3) and agency (Q5) were significantly higher than that in Experiment 3. **(c) Comparison regarding 3PP-hands.** The ratings of the 3PP-hands in Experiment 3 regarding body ownership (Q2), 3PP-subjective tactile location (Q4) and Agency (Q6) were significantly higher than that in Experiment 5. Bold lines indicate the medians; upper and lower limits of the box plot indicate the 75th and 25th percentile. The error bars represent the whole range of the ratings of the statement. Significance levels: *p<0.05; **p<0.01; ***p<0.001.

Abbreviation: STL, subjective tactile location.

III. Remarks on Tsakiris' model of body ownership

Taking the RHI as the main paradigm, Tsakiris' model explains body ownership in terms of three comparisons in multisensory processing. The first concerns visual form congruence: in order for an object, say, a rubber hand, to be felt as part of one's own body, it must look like a human hand[2] (2010, p. 707). The second comparison concerns the postural and anatomical congruence: if the posture of the seen rubber hand is incongruent with the subject's unseen real hand, or if the former is situated too far away from the latter, the RHI would be abolished[2] (pp. 707-708). The third comparison concerns the integration of multisensory inputs related to touch referral[2] (p. 708). These comparisons indicate the inherent constraints for generating the sense of body ownership. According to Tsakiris, fulfilling all these constraints will generate the phenomenon of touch referral, and then the experience of ownership of the viewed object. We will now comment on this model based the findings of this study.

In the synchronous condition of the Passive two-hand condition, the 1PP-hands passed all three comparisons of Tsakiris' model: (1) they were human hands and hence exhibited visual form congruence; (2) The 1PP-hands were situated in the subject's egocentric reference frame such that they looked like sharing the same postural and anatomical properties with the participant's own hands; and (3) The participants received synchronized visual-tactile stimulations with regard to the 1PP-hands. As a result, most subjects felt as if the tactile sensations that they experienced were located in the 1PP-hands, and they felt as if the 1PP-hands were theirs. So far, the model can nicely explain the illusory sense of owning the 1PP-hands as reported above.

But something in the Passive two-hand condition went beyond Tsakiris' model. Although the participants' hands were stationary, still a weak sense of agency on the 1PP-hands was induced and positively correlated with the sense of body ownership. This suggests that a neurocognitive model of body ownership needs to make room for the role of agency. Some have suggested that body ownership and agency are generated by two independent neural systems[3,4,5]. Here, our finding supports the view that the subjective experiences generated by these systems can influence each other.

In the synchronous condition of Experiment 1, the 1PP-hands passed all three comparisons as well. The 3PP-hands did not pass the third comparison because the effect of 3PP-subjective tactile location was not observed. Also, seeing one's own hands from the 3PP affected how the participant experienced the 1PP-hands, so Tsakiris' model can explain why the sense of ownership of the 1PP-hands was weaker (compared

with the sense of ownership of the 1PP-hands in the Passive two-hand condition) and why there was no four-hand illusion in Experiment 1.

However, our data regarding the ownership of the 3PP-hands presents a problem for Tsakiris' model. Since the 3PP-hands did not pass the second and the third comparisons, the model predicts that the sense of ownership of those hands would not be induced. This prediction was undercut by our data. It is true that, in the RHI study, if the fake hand is placed 180º opposite to the subject, the illusion would not be generated because of posture incongruence[6,13]. However, our previous study on the self-touching illusion showed that if the fake hand is replaced by the subject's own hand, the sense of body ownership can still be triggered such that the subject felt as if he/she was brushing his/her own hand (pp. 5-6)[6]. Therefore, it is possible that posture incongruence can be overcome by a high degree of visual form congruence plus synchronized visual-tactile sensations. This means that the second comparison in Tsakiris' model is not rigid.

Consider the synchronous condition of Experiment 3. We were able to trigger the four-hand illusion when we brought body agency and visual agency into play. The finger movements generated visual agency on all four hands, which matched the body agency that the subjects felt via proprioception. By tapping the index fingers and seeing all four hands performing the same movement, plus synchronous tactile stimulations, there was at least a period of time where the participants felt that both the 1PP- and the 3PP-hands were theirs. This suggests that both body agency and visual agency have influences on the first and the second comparisons in Tsakiris' model. As the results indicated, the subjective feelings of ownership and agency of the 1PP-hands were significantly stronger than that in the passive four-hand condition. Although the 3PP-hands did not pass the second comparison, they exhibited a high degree of visual form congruence. Moreover, the subjects not only felt synchronized visual-tactile sensations but also visual agency and body agency simultaneously. Together, these factors prevailed over posture incongruence, and caused the experience of ownership on the 3PP-hands. Our findings are consistent with various studies: Dummer et al. (2009) demonstrated that the sense of body ownership induced by active movement is stronger than body ownership induced by no movement[7]. As reported by Riemer et al. (2013), the resulting proprioceptive drift was stronger in actively moving RHI than that which resulted from RHI without movement[8]. Finally, Zhou et al. (2015) showed that when the experimenter imitated the fist-clenching movements of the participant, the mirrored hand illusion, i.e., the illusory ownership of the hand viewed from the 3PP, could be induced[9].

IV. Other implications and comparisons

In both Experiments 1 and 3 the distribution of scores regarding the 3PP-hands shows a stronger experience in the asynchronous conditions. We think this can be explained by two features of our experiments: (1) since the 3PP-hands were the participant's own hands, they exhibited the highest degree of visual form congruence. (2) Seeing both the 1PP-hands and the 3PP-hands in one view was a subjectively integrated experience. Hence, the subject's experience of the 3PP-hands was influenced by his/her experience of the 1PP-hands, and vice versa. This mutual influence was much stronger in the synchronous condition than in the asynchronous condition. Let us elaborate.

In our set-ups, since both pairs of hands were spatially close to each other and visually salient via the HMD, it was not likely that the participants might watch one pair of hands tapping the index finger without noticing the other pair performing exactly the same movement. Our questionnaire data show that the participants' experiences of the 3PP-hands were influenced by their experiences of seeing the 1PP-hands, and vice versa. For example, compared with the Passive two-hand condition, the sense of 1PP-hand ownership was weakened by seeing the subjects' own hands from the adopted 3PP in Experiment 1. Now consider Experiment 3. In the asynchronous condition, the tapping of the 1PP-hands that the participants saw (1PP-visual agency) was *inconsistent* with their own finger movements (body agency). So the participants did not feel that the 1PP-hands were their own hands, and hence their experience of the 1PP-hands did not affect (or had only minimal influence on) their experience of the 3PP-hands. Notice that, since the 3PP-hands were the participants' own hands, in the asynchronous condition the tapping of the 3PP-hands that the participants saw (3PP-visual agency) was still *synchronized* with their own finger movements (body agency). This synchrony between 3PP-visual agency and body agency, together with the high degree of visual form congruence of the 3PP-hands, can explain why the statements on the 3PP-hands in the asynchronous condition had high scores. Now, why the relevant scores in the synchronous condition were lower? We think this was mainly because, in addition to synchronous visual-tactile stimulations, the tapping of the 1PP-hands that the participants saw (1PP-visual agency) was *consistent* with their own finger movements (body agency). This induced the illusory sense of 1PP-hand ownership. As the participants saw all four hands in one view, their experience of the 1PP-hands affected the participants' experience of the 3PP-hands such that the relevant scores became lower.

Experiment 1 did not involve agency, but a somewhat similar explanation can still apply. In the asynchronous condition, due to the asynchronous visual-tactile stimulations, the participants did not feel that the 1PP-hands were their own hands, and hence their experience of the 1PP-hands did not affect (or had only minimal influence on) their experience of the 3PP-hands. The high degree of visual form congruence of the 3PP-hands can explain why the scores on the 3PP-hands were higher. In the synchronous condition, the synchronous visual-tactile stimulations induced an illusory sense of 1PP-hand ownership. Although, as mentioned above, this illusory sense of 1PP-hand ownership was weaker than that in the synchronous condition of the Passive two-hand condition, it is still significantly stronger than the asynchronous condition of Experiment 1. The scores on the 3PP-hands were lower in the synchronous condition because the participants' experience of the 3PP-hands was influenced by their experience of the 1PP-hands.

We will end this section with a final comparison. Using immersive virtual reality (IVR) techniques, Slater et al. (2010) reported that the induced illusory body ownership from the 1PP was significantly higher than that from the 3PP[10]. But there are a few differences between this study and ours. First, while in Slater et al.'s study the subjects saw virtual body in an IVR environment, the subjects in our Experiment 3 saw real hands tapping in the same pattern. Second, Slater et al.'s study involved only visual agency of the virtual body but not participant's active hand movement. Third, in Slater et al.'s experiment, the participant saw the virtual body either from the 1PP or from the 3PP, but not both at the same time. In contrast, in the synchronous condition of our Experiment 3, the participant saw both the 1PP-hands and the 3PP-hands tapping together synchronously. These differences can explain why in Experiment 3 the induced ownership experiences of both pair of hands were roughly the same (Suppl. figure 4b, Q1 and Q2). Since the set-ups were quite different, the results from these two studies do not necessarily contradict with each other.

REFERENCES

1. Kalckert, A. & Ehrsson, H. H. The moving rubber hand illusion revisited: Comparing movements and visuotactile stimulation to induce illusory ownership. *Conscious. Cogn.* **26,** 117–132; doi:10.1016/j.concog.2014.02.003 (2014).
2. Tsakiris, M. My body in the brain: A neurocognitive model of body-ownership. *Neuropsychologia* **48,** 703–712; doi:10.1016/j.neuropsychologia. 2009.09.034 (2010).
3. Tsakiris, M., Longo, M. R. & Haggard, P. Having a body versus moving your body: Neural signatures of agency and body-ownership. *Neuropsychologia* **48,** 2740–

2749; doi:10.1016/j.neuropsychologia.2010.05.021 (2010).

4. Tsakiris, M., Schuetz-Bosbach, S. & Gallagher, S. On agency and body- ownership: Phenomenological and neurocognitive reflections. *Conscious. Cogn.* **16,** 645–660; doi:10.1016/j.concog.2007.05.012 (2007).
5. Kammers, M. P. M., van der Ham, I. J. M. & Dijkerman, H.C. Dissociating body representations in healthy individuals: Differential effects of a kinaesthetic illusion on perception and action. *Neuropsychologia* **44,** 2430–2436; doi:10.1016/j.neuropsychologia.2006.04.009 (2006)
6. Liang, C., Chang, S. Y., Chen, W. Y., Huang, H. C. & Lee, Y. T. Body ownership and experiential ownership in the self-touching illusion. *Front. Psychol.* **5,** 1591; doi:10.3389/fpsyg.2014.01591 (2015).
7. Dummer, T., Picot-Annand, A., Neal, T. & Moore, C. Movement and the rubber hand illusion. *Perception* **38,** 271–280; PMID:19400435, doi:10.1068/p5921 (2009).
8. Riemer, M., Kleinbohl, D., Holzl, R. & Trojan, J. Action and perception in the rubber hand illusion. *Exp. Brain Res.* **229,** 383–393; PMID:23307154, doi:10.1007/s00221-012-3374-3 (2013).
9. Zhou, A., Zhang, Y., Yin, Y. & Yang, Y. The Mirrored Hand Illusion: I Control, So I Possess? *Perception* **44,** 1225–1230; doi:10.1177/ 0301006615596902 (2015).
10. Slater, M., Spanlang, B., Sanchez-Vives, M. V. & Blanke, O. First Person Experience of Body Transfer in Virtual Reality. *PLoS One* **5,** e10564; doi:10.1371/journal.pone.0010564 (2010).
